# Highly Metastatic Subpopulation of TNBC Cells Has Limited Iron Metabolism and Is a Target of Iron Chelators

**DOI:** 10.3390/cancers15020468

**Published:** 2023-01-12

**Authors:** Yuze Wang, Toshiaki Ohara, Yuehua Chen, Yusuke Hamada, Chunning Li, Masayoshi Fujisawa, Teizo Yoshimura, Akihiro Matsukawa

**Affiliations:** Department of Pathology and Experimental Medicine, Faculty of Medicine, Dentistry and Pharmaceutical Sciences, Okayama University, Okayama 700-8558, Japan

**Keywords:** triple-negative breast cancer, iron metabolism, iron chelator, phosphoinositide-3-kinase-protein kinase, heterogeneity, metastasis

## Abstract

**Simple Summary:**

Excess iron is known to be a risk factor of carcinogenesis. Although iron chelators show anti-cancer effects, they have not been used successfully to treat cancer patients. Triple-negative breast cancer (TNBC) is a disease with poor prognosis without effective treatments. Thus, we aimed to evaluate the possibility of iron chelators as a therapy for TNBC. Deferasirox (DFX), an iron chelator, suppressed the growth of 4T1 murine TNBC cell line cells in vitro and in vivo lung metastatic model. We found that highly metastatic TNBC cells have limited iron metabolism and can be more effectively targeted by iron chelators.

**Abstract:**

Excess iron is known to be a risk factor of carcinogenesis. Although iron chelators show anti-cancer effects, they have not been used successfully to treat cancer patients. Triple-negative breast cancer (TNBC) is a disease with poor prognosis without effective treatments. Thus, we aimed to evaluate a possibility of iron chelators as a therapy for TNBC. Deferasirox (DFX), an iron chelator, suppressed the growth of 4T1 murine TNBC cell line cells in vitro and in vivo. Lung metastasis was further significantly reduced, leading to the hypothesis that iron metabolism between metastatic and non-metastatic cells may be different. An analysis of existing database demonstrated that the expression of iron-uptake genes was significantly suppressed in TNBC cells that metastasized to lymph nodes or lungs compared to those in primary tumors. A highly metastatic clone of the murine 4T1 TNBC cells (4T1-HM) did not proliferate well under iron-rich or iron-depleted conditions by iron chelators compared to a low-metastatic clone (4T1-LM). Bulk RNA-seq analysis of RNA from 4T1-HM and 4T1-LM cells suggested that the PI3K-AKT pathway might be responsible for this difference. Indeed, DFX suppressed the proliferation via the AKT-mTOR pathway in 4T1-HM and the human MDA-MB-231 cells, a human mesenchymal-like TNBC cell line. DFX also suppressed the growth of 4T1-HM tumors in comparison to 4T1-LM tumors, and reduced lung metastases after surgical resection of primary 4T1 tumors. These results indicated, for the first time, that highly metastatic TNBC cells have limited iron metabolism, and they can be more effectively targeted by iron chelators.

## 1. Introduction

Iron is essential to cell proliferation and energy metabolism. However, excess iron is known to be a risk factor of carcinogenesis, tumor progression, and metastasis [1,2,3]. In particular, high heme iron intake increases the risk of carcinogenesis in breast cancer [4]. Thus, iron reduction is logically expected to be a therapeutic approach for cancer, and iron chelators have been investigated as a potential therapeutic agent. Some iron chelators have been shown to inhibit the proliferation of cancer cells, either alone or in combination with other anti–cancer medications [5,6,7,8]. They have also been reported to inhibit the stemness of CSCs [9,10].

Breast cancer is considered the most prevalent cancer in women worldwide, affecting almost 12% of all women during their lifetime [11]. Triple–negative breast cancer (TNBC) is characterized by the absence of estrogen receptor (ER), progesterone receptor (PR), and HER2 (ERBB2) expression, and it is known for its aggressiveness and poor prognosis [12]. High–proliferation or high–grade TNBCs are associated with poor prognosis and rapid progression to the metastatic stage. Moreover, TNBC is a highly heterogeneous tumor type, causing difficulty in the treatment of TNBC [13]. A number of new attempts on immunotherapy and molecular targeted therapy have been conducted [14], however, preoperative neoadjuvant chemotherapy and postoperative chemotherapy and radiotherapy continue to be the common treatments for TNBC [15]. Therefore, TNBC treatment is urgently required, particularly to prevent the progression from local stage to metastatic stage.

Our previous study revealed that iron depletion diet and iron chelators suppressed tumor growth in some kinds of cancer [7,8]. We also revealed that iron chelator possibly inhibited the stemness of cancer [9,16,17]. Furthermore, the possibility using iron chelators for TNBC has been reported [18]. However, the relationship between TNBC metastasis and iron metabolism remains unclear. In this study, we examined the difference in iron metabolism between highly metastatic and low–metastatic subtypes of TNBC cells, and evaluated the effectiveness of iron chelators as a therapeutic modality for the treatment of TNBC.

## 2. Materials and Methods

### 2.1. Cell Culture

The 4T1 murine breast TNBC cell line and human TNBC cell line (MDA–MB–231 and HCC–1937) were acquired from American Type Culture Collection (Manassas, VA, USA). All parent cells and 4T1 single clones were grown in RPMI 1640 medium (Sigma–Aldrich, St. Louis, MO, USA), supplemented with 10% fetal bovine serum (Cytiva, Marlborough, MA, USA), 100 U/mL of penicillin, and 100 g/mL of streptomycin (Sigma–Aldrich) at 37 °C in humidified air with 5% CO_2_.

### 2.2. Reagents

DFX (EXJADE) and deferoxamine (DFO; Desferal) were obtained from Novartis Pharma (Tokyo, Japan). Super–polyphenol 10 (SP10) was kindly provided by Disease Adsorption System Technologies Co., Ltd. (Kanazawa, Japan). For in vitro studies, DFX was dissolved in DMSO (Sigma–Aldrich) at a stock concentration of 50 mmol/L. For in vivo studies, DFX was dissolved in saline (Otsuka Pharmaceutical, Tokyo, Japan). DFO and SP10 were dissolved in distilled water at a stock concentration of 50 mmol/L. The standard iron stock solution Fe^3+^ and Fe^2+^ (Catalog No. 19433–02 and 19505–75; Nacalai tesque, Kyoto, Japan) was dissolved in distilled water at a stock concentration of 50 mmol/L. Ready–made Sirolimus solution (Catalog No. R8781; Thermo Fisher Scientific, Waltham, MA, USA) was diluted with DMSO in different concentrations before any treatment. 

### 2.3. Cell Proliferation Assay

Cell proliferation was evaluated using the XTT assay (Cell Proliferation kit II, Roche, Mannheim, Germany) according to the manufacturer’s instructions. All cell lines were seeded (4.0 × 10^3^/well) in 96–well plates and treated for 48 h with iron chelators or iron reagents with 3% fetal bovine serum. The XTT reagent mixture was added to each well according to the manufacturer’s instructions for 2 h incubation at 37 °C, and 5% CO_2_ in a humidified incubator, then each well was measured using a microplate reader. The experiments were performed three times. Cell viability was calculated and IC_50_ was determined by GraphPad prism 9.0 software.

### 2.4. Western Blotting

After 48 h incubation with medium and specific reagents, whole cells were subjected to protein extraction. For total protein isolation, cells were lysed in lysis buffer (Cell Signaling Technology, Danvers, MA, USA) containing a protease inhibitor cocktail (Roche Life Science) and Halt™ Phosphatase inhibitor cocktail (Thermo Fisher Scientific, Waltham, MA, USA), incubated on ice for 30 min, and centrifuged for 10 min at 14,500× *g*. BCA Protein Assay Kit was used to measure protein concentrations (TaKaRa, Kusatsu, Japan). Equal amounts of protein samples were denatured at 100 °C for 10 min with 4× NuPAGE LDS sample buffer (Invitrogen, Waltham, MA, USA), and 10× Sample Reducing agent (Invitrogen, Waltham, MA, USA). 15–30 μg of cell lysates were separated on 4–12% NuPAGE Bis–Tris precast gel (Thermo Fisher Scientific) and transferred to nitrocellulose blotting membranes (GE Healthcare Life science, Chicago, IL, USA). The membranes were blocked for 40 min at room temperature with 5% skim milk in tris–buffer saline–Tween 20 (TBS–T). Each primary antibody was diluted according to the manufacturer’s instructions. After an overnight incubation with a primary antibody at 4 °C, the membrane was washed with TBS–T three times for 10 min, then incubated with horseradish peroxidase–conjugated secondary antibodies at room temperature for 1 h. After incubation with a secondary antibody, the membranes were washed three times for 10 min, and the presence of the target protein was quantified using a C–DiGit Blot scanner (LI–COR Biosciences, Lincoln, NE, USA). Table S1 enumerates all antibodies used in this study.

### 2.5. Sphere–Formation Assay

For spheroid generation, 5 × 10^2^ cells were seeded into ultra–low attachment 96 well round–bottomed plates (Corning, New York, NY, USA) containing sphere–forming medium DMEM/F12 (Invitrogen, Waltham, MA, USA) supplemented with 1× B27 serum substitute (Invitrogen), 20 ng/mL of mouse recombinant epidermal growth factor (PeproTech, Cranbury, NJ, USA), and 10 ng/mL of basic fibroblast growth factor (PeproTech). The DFX and DFO treatment groups were exposed to 20 μmol/L of DFX and 50 μmol/L of DFO, respectively. After 6 days of using Image J, the length of the radius of spheroids was examined. The volume (μm^3^) of each spheroid was calculated using the following formula: V = πR^3^4/3.

### 2.6. RNA–Seq Assay

In six–well plates, 4T1–HM or 4T1–LM cells were grown for 48 h with or without 50 μmol/L of DFX. Then, the cell’s total RNA was isolated using the High Pure RNA Isolation Kit (Roche Life Science). The Genetic Analysis company (Rhelixa, Tokyo, Japan) performed whole–transcriptome sequencing (Bulk RNA–seq) utilizing the NEBNext^®^ Poly(A) mRNA Magnetic Isolation Module (New England Biolabs, Ipswich, Massachusetts) and NEBNext^®^ UltraTMII Directional RNA Library Prep Kit (New England Biolabs, Ipswich, MA, USA). After depletion of ribosomal RNA (rRNA), conventional Illumina techniques were performed to generate sequencing libraries, which were then processed on an Illumina NovaSeq 6000 sequencing machine (150–bp paired–end reads, two samples per lane; Illumina, San Diego, CA, USA). Raw read data were subjected to quality control using FastQC (accessed on 9 January 2023, https://www.bioinformatics.babraham.ac.uk/projects/fastqc/), and sequence reads were trimmed using Trimmomatic (accessed on 9 January 2023, http://www.usadellab.org/cms/?page=trimmomatic). Sequence reads were aligned with the murine transcriptome (mm10, Ensembl.org) with HISAT2 (accessed on 9 January 2023, http://daehwankimlab.github.io/hisat2/). Statistical significance was established utilizing the R environment with edgeR packages. In reducing false positives, only genes with a *p*–value adjusted to less than 0.05 were considered differential. The data discussed in this publication are accessible through GEO series accession number GSE218133 (accessed on 9 January 2023, https://www.ncbi.nlm.nih.gov/geo/query/acc.cgi?acc=GSE218133).

### 2.7. Database Analysis

**Figure 1:**
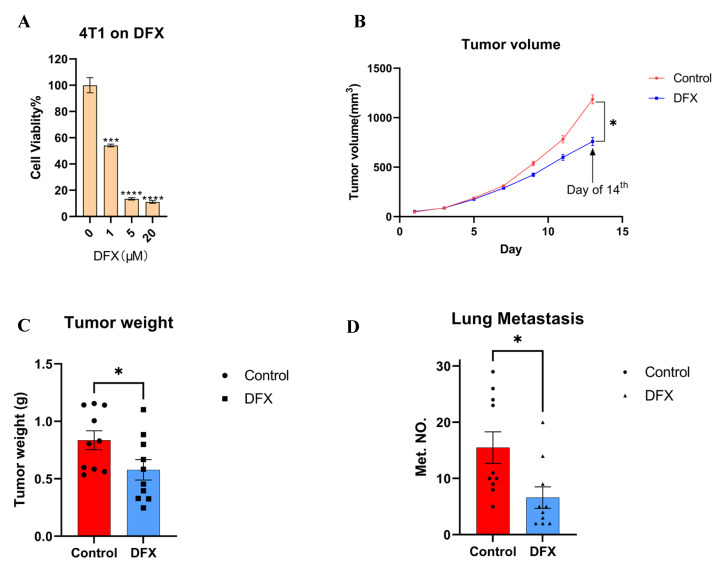
DFX inhibits the growth of 4T1 primary and lung metastatic tumor. (**A**) Murine TNBC cells 4T1 were treated for 48 h with iron chelator DFX with different concentrations (*n* = 6). Cell viability was determined via Cell Proliferation Kit II (XTT). Cell viability in the absence of treatment was set at 100%. Treatment groups were compared with Control group, respectively. The Student’s *t* test with Welch’s correction was utilized to compare two groups, *** *p* < 0.001, **** *p* < 0.0001 and Data shown as the mean ± S. E. M. (**B**) 4T1 cells (1 × 10^5^) were injected in left mammary gland of BALB/c female mice (6–8 weeks old). After 7 days, mice were randomly divided into two groups (*n* = 10), and treatment was initiated as indicated. DFX and Ctrl treated groups were treated with 160 mg/kg DFX or equivalent volume of saline via oral gavage. DFX effectively inhibited the growth of 4T1. At the day of 14th, the tumor volumes were abstracted, and the Student’s *t* test was utilized to compare two groups, * *p* < 0.05 (**C**) Tumor weight was measured in the end of study. DFX effectively decreased tumor weight. The Student’s *t* test was utilized to compare two groups, * *p* < 0.05 (**D**) Lung tissue were collected, and metastatic tumor numbers were counted under stereomicroscope. DFX effectively decreased the number of 4T1 lung metastasis. The Student’s *t* test was utilized to compare two groups, * *p* < 0.05.

We selected the lung metastasis RNA sequence and compared them to the RNA–seq data of primary lesions based on the description in the article [19]. The GSE63180 data were retrieved from GEO datasets (accessed on 9 January 2023, https://www.ncbi.nlm.nih.gov/geo/query/acc.cgi?acc=GSE63180), and the obtained “GSE63180 All Clones Normalized.txt.gz” file was initially processed using R, then R package limma for differential analysis [20]. The metastatic lung tumor and primary tumor of GSE63180 were abstracted and fitted using a linear model, based on which moderated t–statistics, moderated F–statistics, and log–odds of differential expression by empirical Bayes moderation of the standard errors towards a global value were calculated. The threshold for differentially expressed genes was the absolute log2FC value > 0.5 and an adjusted *p*-value < 0.05. GraphPad prism 9.0 was used to produce a graph of iron–related genes (Figure 1A).

### 2.8. Tumor Allograft Model

The experimental protocols of this study were approved by the Animal Care and Use Committee of Okayama University, Okayama, Japan (OKU–2021700), and all experiments were conducted in compliance with applicable policies and procedures.

### 2.9. Orthotopic Tumor Model

Female BALB/c wild–type mice were purchased from Japan SLC, Inc. (Hamamatsu, Japan) and housed in sterile conditions. The experiments began when the mice were 8 weeks old. 4T1 tumor cells in culture were harvested and re–suspended in phosphate–buffered saline. Viable 4T1 cells (1 × 10^5^) were injected into the left thoracic mammary gland. Tumor size and body weight were measured every 3 days for 21 days. DFX (160 mg/kg) was suspended in saline and administered orally using a probe 3 days/week. Treatment started 3 days after tumor implantation (*n* = 10). The control group received saline administered by oral gavage 3 days/week. Tumor volume was calculated using the following formula: Volume = (width)^2^ × length/2. At the end of the experiment, the animals were sacrificed, and the tumors were collected for weight measurement and histological analysis. Bouin’s solution (FUJIFILM Wako Pure Chemical Corporation) was used to perfuse the lungs, which were then fixed in the same solution. The number of metastatic tumors on the lungs was counted under a stereomicroscope.

### 2.10. Single–Cell RNA Sequencing Analysis

Based on the files provided by the GEO dataset database (GSE123837), raw FPKMs for HCI001, HCI002, and HCI010 from three different patients were extracted to generate a gene expression matrix. We eliminated cells with less than 2500 expressed genes, a proportion of mitochondrial genes that exceed 50% of all expressed genes, or a proportion of potential erythrocyte genes that exceed 0.025% of all expressed genes. A total of 1707 filtered cells were utilized for additional bioinformatic analysis. Seurat package version 4.1.1 was used to merge the three data frames. Normalize Data (object = object, normalization.method = “LogNormalize”) and Find Variable Features (object = object, selection.method = “vst,” nfeatures = 10,000) were utilized to normalize the data. After normalizing the data and setting Default Assay to integrated, we combined all three patient–derived cell datasets using Find Integration Anchors (object.list = object.list, dims = 1:30, anchor.features = 10,000) and Integrate Data (anchorset = anchors, dims = 1:30). Then, we centralized the data using the formula Scale Data (object, vars.to.regress = c(“S.Score,” “G2M.Score”)) to scale out the influence of cycling genes on clustering.

Seurat analysis package version 4.1.1 was used to perform dimensionality reduction and differential gene expression. Then, a principal component analysis was performed using variable genes. The data were subjected to UMAP using the produced principal components. Seurat’s Find Clusters function and a granularity parameter of 0.8 were also used to identify each cluster for the analysis of merged data.

In addition, we utilized the R package AUCell to evaluate pathway activities of specific cells. The AUCell_buildRankings function was used to compute the gene expression rankings in each cell using an expression matrix and default parameters. Reference articles were used to compile the iron–uptake gene set. The gene set was used to assign scores to each cell, and AUC values were calculated for the gene set and cell using the AUCell_calcAUC function. These AUC values represented the percentage of genes within the top–ranking genes for each cell that belonged to the pathway gene set.

### 2.11. Statistical Analysis

GraphPad prism 9.0 was used to analyze the data, and the results were presented as the mean (standard error of the mean). Each experiment was technically and biologically in triplicate. Student’s *t* test was utilized to compare two groups, and *p* < 0.05 was considered statistically significant. F–test was utilized for variance analysis between two groups. When *p* < 0.05 in the F–test, the *t* test with Welch’s correction was used to compare two groups, *p* < 0.05 was determined statistically significant.

## 3. Results

### 3.1. Iron Chelators Suppressed In Vitro Cell Proliferation, In Vivo Tumor Growth, and Lung Metastasis in a Murine 4T1 TNBC Model

First, murine 4T1 TNBC cell line cells were used to evaluate the anti–cancer effect of iron chelators. Iron chelators, including deferasirox (DFX), deferoxamine (DFO), and super–polyphenol 10 (SP10), dose–dependently suppressed the proliferation of 4T1 cells in vitro (Figure 1A; Supplemental Figure S1A,B). As DFX with the lowest IC_50_ for 4T1 cells (DFX: 1.083 μM; DFO: 8.175 μM; SP10: 95.199 mM), DFX had the most suppressive effect compared to the others. To verify the effect of DFX in vivo, a 4T1 orthotopic allograft model was used. DFX suppressed the primary tumor growth by approximately 30% (Figure 1B,C). Furthermore, DFX suppressed lung metastasis by more than 50%, which was a more evident effect compared to the effect on primary tumor growth (Figure 1D; Supplemental Figure S1C). Body weights of treated mice were not significantly different (Supplemental Figure S2). Based on the results, we hypothesized that highly metastatic TNBC cells are more sensitive to iron chelators due to different iron metabolism and iron chelators could be a therapeutic modality of breast cancer.

### 3.2. Database Analysis Showed That the Iron–Uptake Genes Are Suppressed in a Highly Metastatic TNBC Cell Patient–Derived Xenograft (PDX) Model and 4T1 Cells

**Figure 2:**
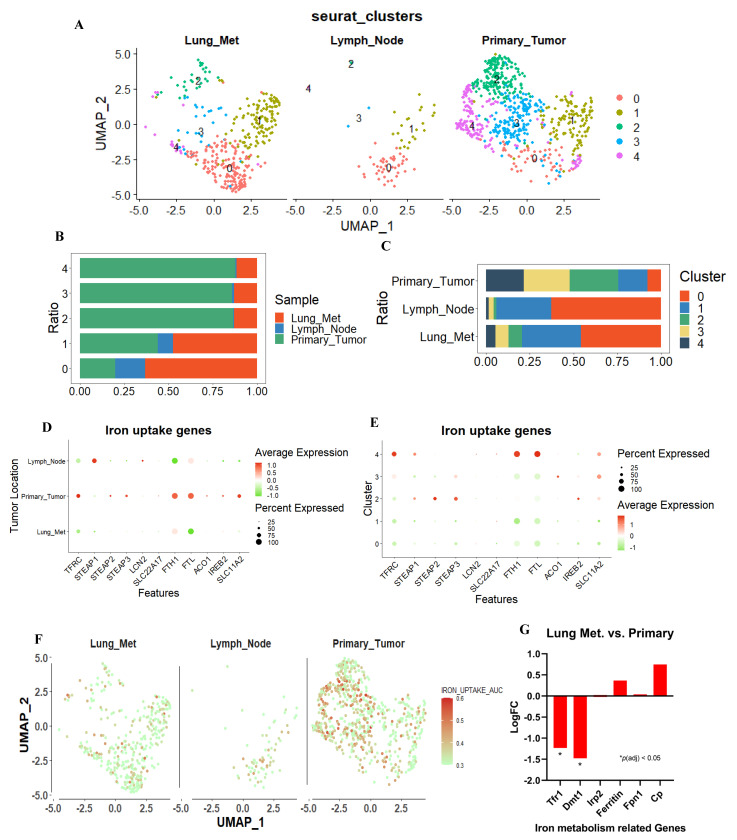
scRNA–seq analysis for TNBC–PDX tumor cells. (**A**) Uniform Manifold Approximation and Projection (UMAP) showing clustering of 1108 metastatic and primary tumor cells from the merged comprehensive data. Lung metastasis, lymph node metastasis, and primary tumor revealed different cluster pattern. (**B**,**C**) The proportion of tumor metastases and primary tumors within each cell cluster, and the proportion of tumor metastases and primary tumors within each cell cluster. (**D**,**E**) Dot plots were used to visualize iron–uptake genes, with the size of the dot representing the percentage of gene–expressed cells and the color representing the expression level of each assassinated group, with higher scores nearer red and lower scores nearer green. (**F**) The AUC value for each cell was calculated using “Iron uptake gene set”, and UMAP coordinates were used to display the position of each cell in order to visualize the AUC scores for this gene set per cell, with higher scores closer to red and lower scores closer to green. (**G**) Iron related genes were extract from 4T1 GEO’s public datasets (GSE63180). Tfr1 and Dmt1, iron intake genes, were significantly suppressed in lung metastasis site compared to primary tumor. * *p <* 0.05.

To explore the difference in iron metabolism between highly metastatic and low metastatic TNBC cells, the single–cell mRNA sequence database based on TNBC PDX model (GSE123837) was analyzed. Single cells were obtained from primary and metastatic tumors, and their metabolic functions were analyzed. We also examined the difference in biological functions using the same dataset. First, we imported the RNA–seq FPKM data of 1707 cells from GSE123837 and filtered out low–quality cells containing fewer than 300 gene features. We selected 1108 cells with more than 2500 gene features, less than 50% of mitochondrial genes, and less than 0.025% of erythrocyte genes, then filtered out abnormal or substandard cells (Supplemental Figure S3). Unbiased cell clustering based on uniform manifold approximation and projection (UMAP) investigations revealed five primary clusters in parallel, in accordance with their gene profiles. Unsupervised clustering separates all cells with the same biological properties into each cluster, where each cluster represents a distinct gene expression pattern or biological trait. (Figure 2A). We observed that the cells from the primary tumor and metastases have distinct biological characteristics. In lung and lymph node metastases, the proportion of clusters 0 and 1 was greater than 75%, and the proportion of cluster 0 originating from lung metastases was greater than 60% (Figure 2B,C). Therefore, unbiased clustering can effectively interpret the gene expression profile of metastases and primary sites.

Next, we extracted a group of genes associated with iron–uptake and examined the differences between primary and metastatic lesions. First, we evaluated genes that were associated with iron–uptake. Dot plots were generated for each gene, revealing that iron intake genes were primarily upregulated in the primary tumor and downregulated in the metastatic tumor (Figure 2D). When viewed from the perspective of the cluster, iron–uptake genes were expressed at a low level in clusters 0 and 1, which accounted for the majority of metastases. On the contrary, they were strongly expressed in clusters 2, 3, and 4, which comprised the majority of primary tumor (Figure 2E). We compiled these genes into a gene set that is associated with iron–uptake, and assessed the overall expression level of this gene set in each cell by calculating the area under the curve (AUC), which identifies cells with active gene signatures regarding iron–uptake gene set in single–cell RNA–seq data. The distribution of AUC values across all cells allows for an examination of the expression of signature gene in relation to one another. Consistent with our hypotheses, genes associated with iron–uptake were strongly expressed in the primary tumor but weakly expressed in the metastatic tumors (Figure 2F). Furthermore, to confirm the trend in murine TNBC, GEO public datasets (GSE63180) were used to identify the difference in iron metabolism–related transcriptomes between primary 4T1 tumor and metastases. The expression of Tfrc1 and Dmt1, two iron transporter genes, was significantly suppressed in the metastatic tumor among all related genes in the 4T1 model, and in the PDX model (Figure 2G). These results indicated that the expression of iron–uptake genes was suppressed in highly metastatic TNBC subpopulations in the human TNBC PDX model and the murine TNBC model.

### 3.3. Tolerance of Iron Metabolic Change Differs between Metastatic and Primary Sites in Murine TNBC Cells

**Figure 3:**
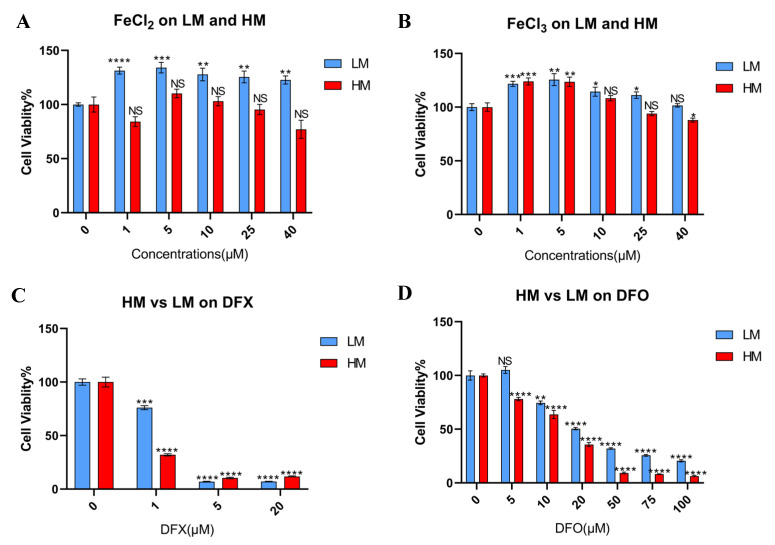
The tolerance of iron metabolic change differs between metastatic and primary sites in 4T1 cells. (**A**,**B**) Murine TNBC cells, 4T1–HM, and 4T1–LM cells were treated for 48 h with iron additional condition by FeCl_2_ and FeCl_3_ (*n* = 6). (**C**,**D**) 4T1–HM and 4T1–LM were treated for 48 h with iron chelators. DFX and DFO were used (*n* = 6). Cell viability was determined via Cell Proliferation Kit II (XTT). Cell viability in the absence of treatment was set at 100%. Treatment groups were compared with Control group, respectively. The Student’s *t* test with Welch’s correction was utilized to compare two groups, NS represented not significant, * *p* < 0.05, ** *p* < 0.01, *** *p* < 0.001, and **** *p* < 0.0001, and Data shown as the mean ± S. E. M.

To confirm the result of database analysis, two 4T1 single cell clones with a high and low metastatic capacity (4T1–HM and 4T1–LM, respectively) were used (Supplemental Figure S4A) [21]. Iron–rich and iron–depleted conditions were made by ferrous and ferric iron compounds and three iron chelators, to explore the effects of an iron metabolic change. In general, cancer cells are enhanced to proliferate in an iron–rich condition. However, the proliferation of 4T1–HM cells was not enhanced under a ferrous or ferric iron–rich condition (Figure 3A,B). Furthermore, 4T1–HM cells were more intolerable than 4T1–LM under iron–depleted conditions created by DFX, DFO, and SP10 (Figure 3C,D; Supplemental Figure S4B). We found that 4T1–HM cells are more sensitive to changes in iron concentration. These results were consistent with the results of database analysis.

### 3.4. PI3K–AKT Pathway Was Responsible for Metastatic Phenotype and Strongly Inhibited by DFX in 4T1–HM Cells

**Figure 4:**
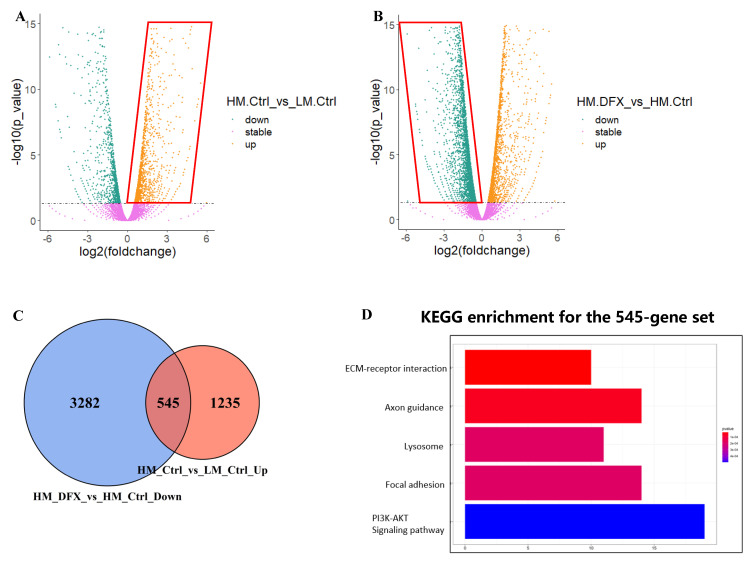
Bulk RNA–seq data analysis shows that DFX more notably inhibits 4T1–HM than 4T1–LM by specifically inhibiting Pi3k–Akt pathway. (**A**,**B**) 4T1–HM and 4T1–LM cells were treated for 48 h with DFX (50 μmol/L) or with equivalent DMSO alone, nominated as HM_Ctrl, LM_Ctrl, HM_DFX, and LM_DFX. Four groups of samples were performed with bulk RNA–seq assay. The different expression gene sets were presented with volcano plots including the different gene expression of HM_Ctrl vs. LM_Ctrl and HM–DFX vs. HM_Ctrl. (**C**) The upregulated gene set in (**A**), and the downregulated gene set in (**B**) were merged with as presented on Venn graph. (**D**) KEGG enrichment was performed with the merged gene set, which showed that Pi3k–Akt pathway might be a potential mechanism underlined DFX more predominant inhibitory effects on 4T1–HM cell. Different expression genes were thought as significant change with a parameter of FDR < 0.05, and logFC > 0 as upregulated otherwise down regulated genes.

RNA sequencing analysis was performed to reveal the mechanism that causes the difference in the tolerance to change in iron concentration between 4T1–LM and 4T1–HM cells. We used RNA from 4T1–LM and 4T1–HM cells either treated or not treated with DFX. We selected DFX because of its stronger inhibitory effect on cancer cell proliferation compared with DFO. We selected genes that were highly expressed in 4T1–HM than in 4T1–LM (Figure 4A). This collection of highly expressed genes primarily can be responsible for metastatic phenotype of 4T1–HM. Consequently, 4T1–HM exhibits distinct growth and metabolic processes. In revealing the effect of iron chelators, downregulated genes in DFX–treated 4T1–HM cells were extracted (Figure 4B). This group of downregulated genes appeared crucial to DFX’s potential to selectively inhibit the proliferation of 4T1–HM. The intersection of the two gene sets was determined (Figure 4C). This newly identified overlapping gene set explains why DFX inhibits 4T1–HM more than 4T1–LM. DFX can selectively inhibit essential genes that are selectively overexpressed in 4T1–HM, which are the key growth and metastasis. To discover the biological mechanism hidden within the newly merged gene set, we used KEGG enrichment (Figure 4D). We found that the PI3K–AKT pathway was significantly upregulated in 4T1–HM cells, then significantly suppressed by DFX. Therefore, we hypothesized that DFX could inhibit metastatic TNBC cells via the PI3K–AKT pathway and performed subsequent step verification.

### 3.5. Upregulated AKT–mTOR Pathway in Metastatic Phenotype Was Strongly Inhibited by DFX

**Figure 5:**
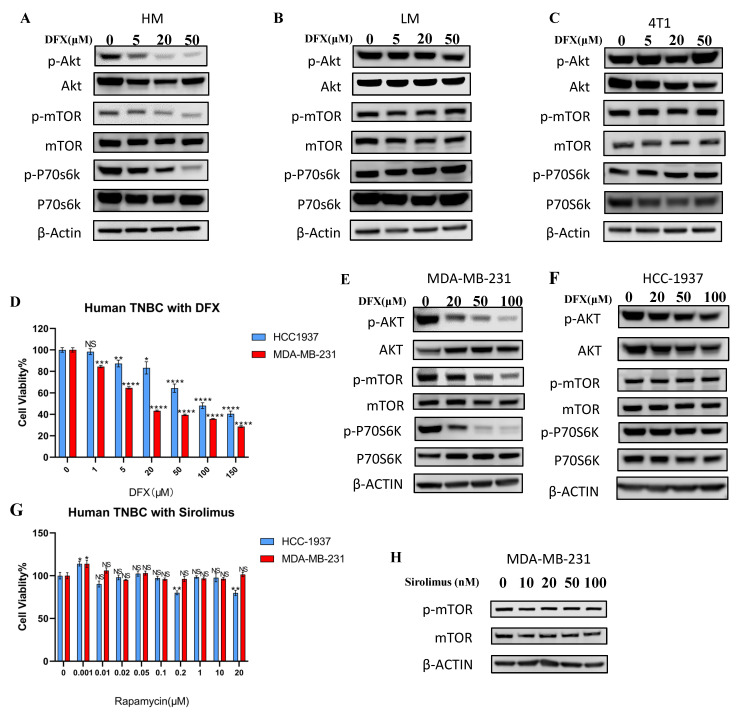
DFX inhibits the AKT/mTOR signaling pathway. (**A**–**C**) 4T1–HM, 4T1–LM, and 4T1 parent cells were treated for 48 h with DFX or with equivalent DMSO alone, followed by the collection of whole cell lysis buffer. The protein expression level was detected by western blot. β–actin was used as an internal control. (**D**) Human TNBC cells, MDA–MB–231, and HCC–1937, were similarly treated for 48 h with DFX (*n* = 6). Cell viability was determined via Cell Proliferation Kit II (XTT). Treatment groups were compared with Control group, respectively. The Student’s *t* test with Welch’s correction was utilized to compare two groups, ** *p* < 0.01, *** *p* < 0.001, and **** *p* < 0.0001, and Data shown as the mean ± S. E. M. (**E**,**F**) MDA–MB–231 and HCC–1937 were treated for 48 h with DFX or with equivalent DMSO alone, followed by the collection of whole cell lysis buffer. The protein expression levels were detected by western blot. (**G**) Human TNBC MDA–MB–231 and HCC–1937 were treated for 48 h with sirolimus in different concentration or with equivalent saline or DMSO alone (*n* = 6). Cell viability was determined via Cell Proliferation Kit II (XTT). Treatment groups were compared with Control group, respectively. The Student’s *t* test with Welch’s correction was utilized to compare two groups, NS represented not significant, * *p* < 0.05 and ** *p* < 0.01, and Data shown as the mean ± S. E. M. (**H**) Human TNBC MDA–MB–231 were treated for 48 h with varying concentrations of sirolimus, followed by the collection of whole cell lysis buffer after 48 h. The protein expression levels of p–mTOR and mTOR were detected by western blot. β–actin was used as an internal control.

Based on the RNA sequencing result, we examined the effect of DFX on the PI3K–AKT–mTOR pathway in 4T1–HM, 4T1–LM, and parental 4T1 cells. The level of p–AKT was higher in 4T1.HM than 4T1.LM without treatment (Supplemental Figure S5, see full western blot images and histogram in Supplementary Materials). DFX markedly suppressed the level of p–AKT, p–mTOR, and downstream p70S6k in 4T1–HM compared with 4T1–LM and parental 4T1 cells (Figure 5A–C, see full western blot images and histogram in Supplementary Materials). To explore the effect of DFX in human TNBC cell lines, MDA–MB–231 (mesenchymal–like mimicking 4T1–HM), and HCC–1937 cells (basal–like mimicking 4T1–LM) were used. The proliferation of MDA–MB–231 was more suppressed by DFX than HCC–1937 (Figure 5D). Phosphorylation of AKT, mTOR, and downstream molecule p70S6k were significantly suppressed by DFX in MDA–MB–231 compared with HCC–1937 (Figure 5E,F, see full western blot images and histogram in Supplementary Materials). Thus, both murine and human TNBC cells with metastatic phenotype of TNBC cells were selectively inhibited by DFX. Moreover, we compared the effects of DFX with those of an established mTOR inhibitor for human, namely, Sirolimus [22]. Similar to previous reports, Sirolimus did not significantly inhibit proliferation and activation of the AKT–mTOR pathway in MDA–MB–231 and HCC–1937 cells (Figure 5G,H, see full western blot images and histogram in Supplementary Materials). These results indicated that DFX is a better inhibitor of mTOR in human TNBC cells than rapamycin. 

### 3.6. Iron Chelators Induce Apoptosis and Inhibit Stemness in 4T1 Cells

**Figure 6:**
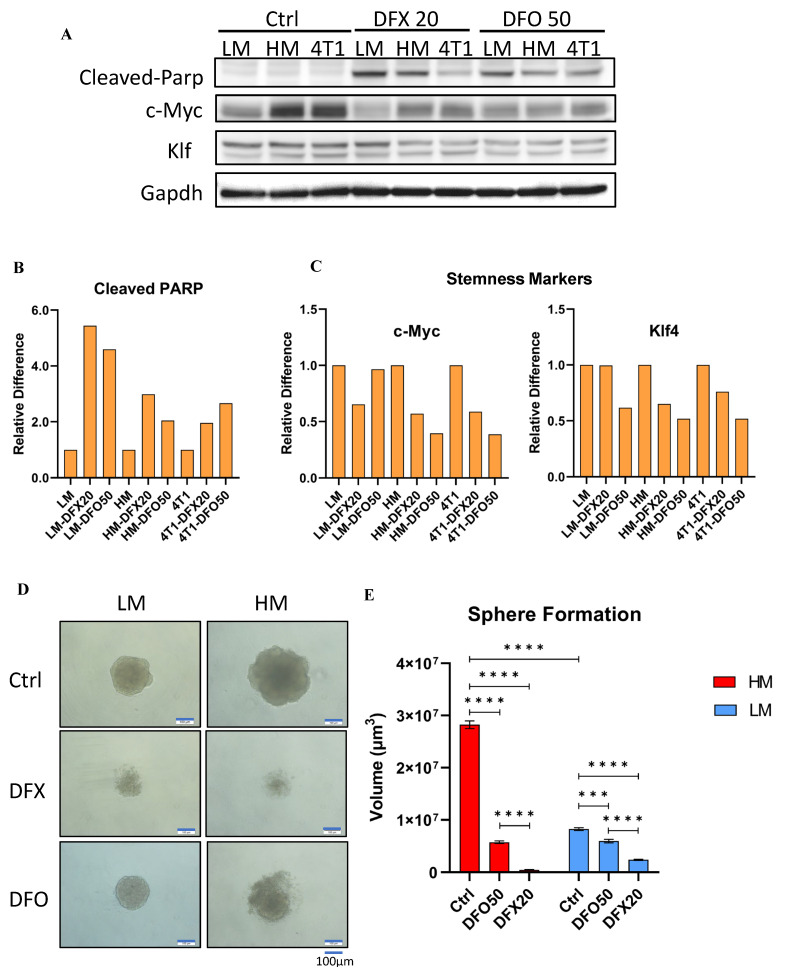
Iron chelators induce apoptosis and inhibit stemness of the 4T1 and its single clones. (**A**) 4T1–HM, 4T1–LM, and 4T1 parent cells were treated for 48 h with indicated reagents (DFX: 20 μmol/L or DFO: 50 μmol/L), or with equivalent DMSO alone. The protein expression levels of Cleaved–Parp, KLF–4, and C–Myc were detected by western blot. (**B**,**C**) The relative expression of each target protein was calculated. Each result was performed with three independent experiments. (**D**) Functional stemness ability was evaluated by sphere formation assay. Sphere formation of 4T1–HM cells was significantly inhibited by iron chelators (DFX: 20 μmol/L or DFO: 50 μmol/L, *n* = 6, scale bar: 100 μm). (**E**) Sphere formed volume was calculated by Image J software. Iron chelators significantly decreased the sphere formed volume. The volume (μm^3^) of each spheroid was calculated using the following formula: V = πR^3^4/3. The Student’s *t* test with Welch’s correction was utilized to compare two groups, *** *p* < 0.001 and **** *p* < 0.0001.

To examine the ability of iron chelators to inhibit stemness, the levels of Cleaved PARP apoptosis marker, and c–Myc and Klf–4 stemness markers were examined in 4T1–HM, 4T1–LM, and parental 4T1 cells (Figure 6A, see full western blot images in Supplementary Materials). The level of Cleaved PARP was increased by DFX and DFO in all cell lines (Figure 6B). c–Myc and Klf–4 levels were strongly suppressed in 4T1–HM cells compared with 4T1–LM cells (Figure 6C). Sphere–formation assay was performed to confirm the inhibition of stemness by iron chelators. DFX and DFO markedly suppressed sphere–formation in 4T1–HM compared with 4T1–LM cells (Figure 6D,E). The effect of DFX was stronger than that of DFO because 20 µM of DFX showed a stronger effect than 50 µM of DFO. These results indicated that iron chelators suppressed TNBC cell proliferation by increasing apoptosis and inhibition of stemness. The effect was more significant in 4T1–HM cells than in 4T1–LM cells probably due to the intolerance of 4T1–HM to iron metabolic change.

### 3.7. DFX Inhibited TNBC Tumor Growth in Orthotopic and Lung Metastatic Models

**Figure 7:**
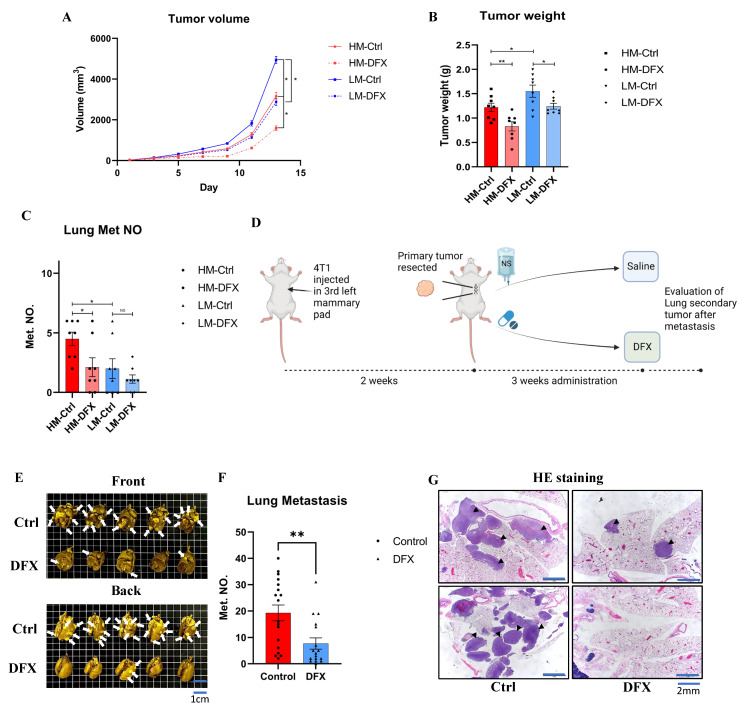
DFX inhibits the growth of 4T1–LM, 4T1–HM, and 4T1 primary and lung metastatic tumor in vivo. (**A**) 4T1–LM (1 × 10^5^) and 4T1–HM cells (1 × 10^5^) were injected in left mammary gland of BALB/c female mice (6–8 weeks old). After 7 days, each group of mice were randomly divided into two groups (*n* = 8), and treatment was initiated as indicated. DFX and Ctrl treated groups were treated with 160 mg/kg DFX or equivalent volume of saline via oral gavage. Tumor volume was measured. The Student’s *t* test was utilized to compare two groups, * *p* < 0.05 and Data shown as the mean ± S. E. M. (**B**) Tumor weight was measured in the end of study. DFX effectively decreased tumor weight. Tumor volume was measured. The Student’s *t* test was utilized to compare two groups, * *p* < 0.05 and ** *p* < 0.01, and Data shown as the mean ± S. E. M. (**C**) Lung tissue were collected, and metastatic tumor numbers were counted under stereomicroscope. Tumor volume was measured. The Student’s *t* test was utilized to compare two groups, * *p* < 0.05. and Data shown as the mean ± S. E. M. (**D**) Diagram shows the experimental model mimicking treatment for lung metastasis after surgical resection of primary tumor. 4T1 cells (1 × 10^5^) were injected in left mammary gland of BALB/c female mice (6–8 weeks old). After 2 weeks, primary tumors were resected and randomly divided into two groups (DFX: *n* = 18; Saline Ctrl: *n* = 17). DFX and Ctrl treated groups were treated with 160 mg/kg DFX or equivalent volume of saline via oral gavage for 3 weeks. (**E**) Lung tissues were collected after sacrifice. DFX decreased the number and size of lung metastasis. White arrow heads indicate lung metastases that are evident (but not every single one). (**F**) Metastatic tumor number were counted under stereomicroscope. DFX decreased the number of 4T1 lung metastasis. Tumor volume was measured. The Student’s *t* test was utilized to compare two groups, ** *p* < 0.01, and Data shown as the mean ± S. E. M. (**G**) Metastasis tumors were performed H and E staining. DFX decreased the number and volume of tumor. Black arrow heads indicate lung metastases that are evident (but not every single one).

An orthotopic model was used to verify the effects of DFX in vivo. DFX significantly suppressed the 4T1–HM tumor growth compared to 4T1–LM (Average reduction rate: HM vs. LM: 32% vs. 20%) (Figure 7A,B). Lung metastasis was also significantly suppressed by DFX in 4T1–HM compared with 4T1–LM (Figure 7C). The body weight of treated mice was not significantly differentiated (Supplemental Figure S6A). 

To evaluate clinical relevance, 4T1 tumors were surgically resected at 2 weeks, then DFX was administered orally for 3 weeks, and the level of lung metastasis was evaluated (Figure 7D). This model could simulate the clinical practice of TNBC treatment No significant difference in tumor weight and body weight were observed before surgery (Supplemental Figure S6B). The number and weight of tumors in the lung were significantly reduced in the DFX–treated group compared to the saline control group (Figure 7E–G). In addition, no significant difference in body weight and no symptomatic adverse effects were observed during the 3 week treatment period, (Supplemental Figure S6C). Based on the results of our in vivo studies, DFX had a therapeutic potential for primary TNBC tumor and preventing effect for lung metastasis.

## 4. Discussion

TNBC is the general term for breast cancers devoid of ER, PR, and HER2 (ERBB2) expression; however, it is characterized by substantial inter–patient heterogeneity. Evidently, an overall examination of this category of breast cancer is insufficient and unconvincing. Therefore, it is necessary to classify and identify treatment options for each subgroup are necessary. Lehmann and colleagues identified six new TNBC subtypes based on gene expression profiles: two basal–like subgroups (basal–like 1 [BL1] and basal–like 2 [BL2]), two mesenchymal subgroups (mesenchymal [M] and mesenchymal stem–like [MSL] subgroups), one immunomodulatory subgroup, and one luminal androgen receptor group [23]. A previous study classified a large number of currently stable cell lines, including MDA–MB–231 as MSL subtype and HCC–1937 as BL1 subtype [23]. Our study also examined the classification and revealed that metastatic TNBC with an activated PI3K pathway has limited iron metabolism by using murine and human TNBC cell lines. Consequently, DFX, an oral iron chelator, was more effective on MSL subtype than on BL1 subtype.

Twenty–five percent of primary TNBCs exhibit mutations or alterations in the PI3K pathway. Evidence showed that abnormal activation of the PI3K pathway facilitates the development of stem cell–like properties and promotes invasive properties and epithelial–to–mesenchymal transition, which is strongly associated with increased invasiveness and metastasis, and resistance to targeted therapy [24]. Patients with high expression of the PI3K/AKT pathway have a poor prognosis, and they will experience earlier metastasis [25]. We found that the PI3K/AKT pathway was responsible for essential growth and metabolism functions of the metastatic phenotype of TNBC, which could be effectively inhibited by DFX. To our knowledge, this study is the first to demonstrate that iron chelators could inhibit highly metastatic cell subsets within TNBC tumors by inhibiting the PI3K/AKT pathway due to limitation of iron metabolism in the subsets. At present, the antitumor effect of inhibitors targeting the PI3K/AKT pathway has been intensively studied, and some of these inhibitors are already undergoing clinical trials and are anticipated to exert anti–TNBC effects in therapy [26]. Our study examined the effect of iron chelators on two human TNBC cell lines, and the results demonstrated that iron chelators inhibited the proliferation and PI3K pathway more intensely in MDA–MB–231 than in HCC–1937. Lehmann and colleagues discovered that the IC_50_ of a PI3K/mTOR inhibitor, NVP–BEZ235, for MDA–MB–231 was lower than that for HCC–1937, and that breast cancers of M and MSL subtypes were more sensitive to PI3K/mTOR inhibitor treatment [23]. MDA–MB–231 was isolated from pleural effusions [27], while HCC–1937 was isolated from a primary breast cancer lesion [28]. Thus, MDA–MB–231 and HCC–1937 were used as MSL and BL1 subtypes, respectively. In addition, other studies have confirmed that MDA–MB–231 is more invasive than HCC–1937 [29]. We assume that MDA–MB–231 has a higher metastatic potential than HCC–1937, and that inter– and intra–tumoral heterogeneity is associated with each other to some extent. The clinical use of the PI3k/mTOR inhibitor NVP–BEZ235 has not been proven, and Rapamycin and its derivatives, which are clinically available, could not provide sufficient effect on human TNBC cell lines in this study. DFX is clinically available and could provide a stronger effect compared to Sirolimus. In the current treatment of human breast cancer, the primary tumor will be resected at an early stage according to the relevant treatment guidelines [11]. In our experiment, the use of DFX for the in vivo 4T1 lung metastatic model with surgical resection of primary tumor is acceptable according to the guidelines.

Research on a relationship between iron and the occurrence and progression of tumors was first proposed in 1959 [30]. Iron is closely associated with the occurrence, progression, and dissemination of malignant tumors. Iron participates in Fenton chemistry; the production of reactive oxygen species and subsequent DNA damage are believed to be the primary mechanisms underlying iron’s carcinogenicity [31]. Excess iron promotes tumor development. On contrary, iron depletion caused by dietary restriction or iron chelators inhibits tumor growth [32,33]. By manipulating the proteins of iron metabolism, tumor growth can also be regulated. Thus, decreasing cellular iron import by inhibiting transferrin and increasing cellular iron export by overexpressing ferroportin (Fpn) inhibit tumor growth [34,35,36,37]. Low expression of Fpn in patients with breast cancer is associated with poor prognosis, whereas high expression of Fpn combined with high levels of Hepcidin can reduce distant metastasis–free survival [38]. Moreover, the 16–gene gene set, known as the iron–regulatory gene signature, is a good predictor of metastasis–free survival in patients [39]. In preclinical studies, the use of iron chelator as therapeutic agents and several iron chelators, differing in structure and pharmacology, has demonstrated antitumor activity through iron depletion [3].

Although we initially discovered that iron chelators can target highly metastatic TNBC cells, the iron metabolism in human is not exactly the same as that in mouse. The type of cancer cell heterogeneity is different among patients and even within each tumor. Our experiment could not cover all types of heterogeneity. In addition, there is no clinical safety data for the use of DFX in patients with TNBC. Therefore, further studies are needed to evaluate the effects of DFX on TNBC.

## 5. Conclusions

During TNBC development, a small subpopulation of cancer cells requires an ability to metastasize to distant organs, such as the lungs. In these cells, the PI3K/Akt/mTOR pathway is activated, resulting in increased cell mobility and stemness; however, these cells limited iron metabolism and became more sensitive for iron chelators. Several inhibitors targeting mTOR, including so–called Rapalogs, have been developed but they may not be effective for TNBC cells as presented here. In contrast, iron chelators effectively inhibited mTOR in metastatic TNBC cells and reduced both the size of the primary tumor and lung metastases in a mouse TNBC model. It would be of great interest to evaluate whether iron chelators can be a useful modality to treat patients with TNBC.

## Data Availability

RNA–seq for 4T1 single clones has been uploaded in GEO database as the accession number: GSE218133.

## Supplementary Materials

The following supporting information can be downloaded at: https://www.mdpi.com/article/10.3390/cancers15020468/s1, Figure S1: Iron chelators DFO and SP10 inhibit primary 4T1 cells. Figure S2: Body weight showed no statistically significant difference at the end of primary tumor study. Figure S3: GSE123837 dataset was retrieved, and general data were displayed in detail and grouped by tumor origin. Accordingly, the number of features, the number of counts, the percentage of mitochondrial genes, and the percentage of erythrocyte genes were listed. Figure S4: Morphology of 4T1–parent cell line and its single clones were displayed. 4T1–HM and 4T1–LM were treated with iron chelators for 48 h. SP10 was used. Cell viability was determined via Cell Proliferation Kit II (XTT). Figure S5: Whole–cell lysis buffer of 4T1–HM, 4T1–LM, and 4T1 parent cells was collected, and the protein expression level was detected by Western blot. β–actin was used as an internal control. Figure S6: Body weight of 4T1–HM and 4T1–LM tumor bearing mice was not statistically significant. Body weight and tumor weight were not statistically significant in surgical mimic model. Table S1: Antibodies applied in this study.

## Abbreviations

BL1	Basal–like 1
BL2	Basal–like 2
CSC	Cancer Stem Cell
DFX	Deferasirox
DFO	Deferoxamine
ER	Estrogen Receptor
Fpn	Ferroportin
M	Mesenchymal
MSL	Mesenchymal Stem–like
PDX	Patient–derived Xenograft
PI3K	Phosphoinositide-3-kinase-protein Kinase
PR	Progesterone Receptor
SP10	Super–polyphenol 10
TNBC	Triple–negative Breast Cancer
UMAP	Uniform Manifold Approximation and Projection
