# Supplementary material for: Highly Metastatic Subpopulation of TNBC Cells Has Limited Iron Metabolism and Is a Target of Iron Chelators
Source: Cancers (Basel). 2023 Jan 12;15(2):468. doi: 10.3390/cancers15020468 (PMC9856351; doi:10.3390/cancers15020468)
Supplement: Supplementary file 1 [file cancers-15-00468-s001.zip › Supp Figures.pdf]

Sup.
Figure S1

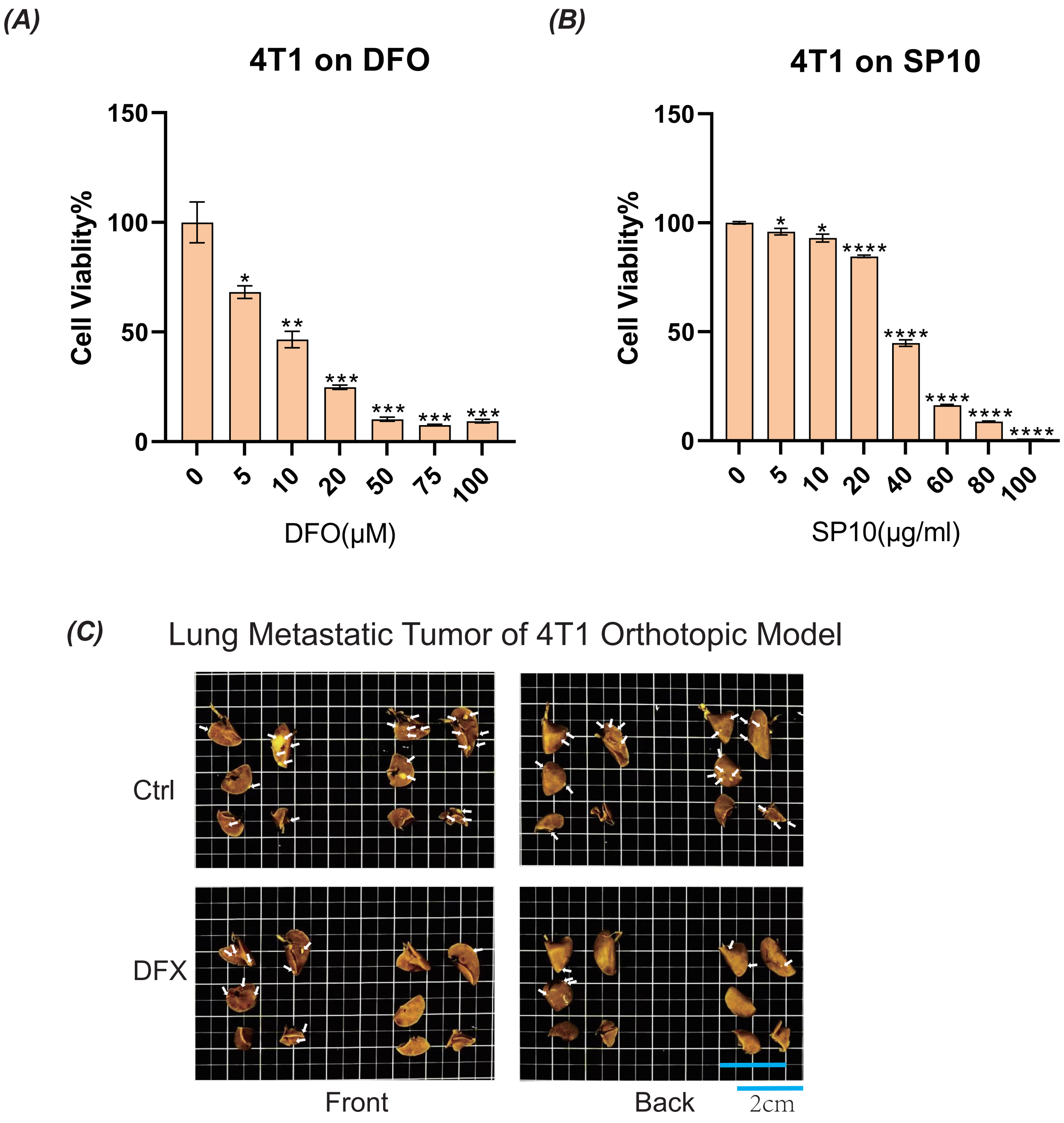

Supplemental Fig. S1 Iron chelator DFO and SP10 inhibit primary 4T1 cells. (a, b) Murine TNBC 4T1 cells were treated for 48h with DFO and SP10. Cell viability was determined via Cell Proliferation Kit II (XTT). Cell viability in the absence of treatment was set at 100%. The student's t test with Welch's correction was utilized to compare two groups, p***<.001, p****<.0001 and Data shown as the mean ± S. E. M. (c) Lung tissue were collected after sacrifice 4T1 orthotopic allograft mice. DFX decreased the number and size of lung metastasis. White triangles signify lung metastases that are evident (but not every single one).

Sup.
Figure S2

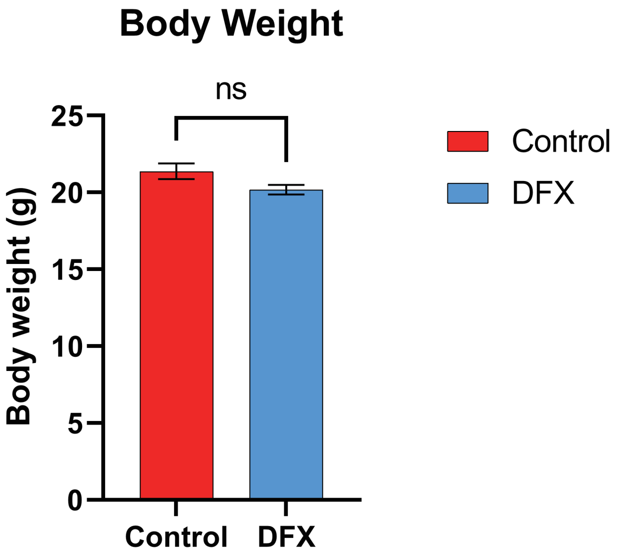

Supplemental Fig. S2 Body weight of two groups were compared with the student's t test, NS represented not significant, and Data shown as the mean ± S. E. M.

**Sup.
Figure S3**

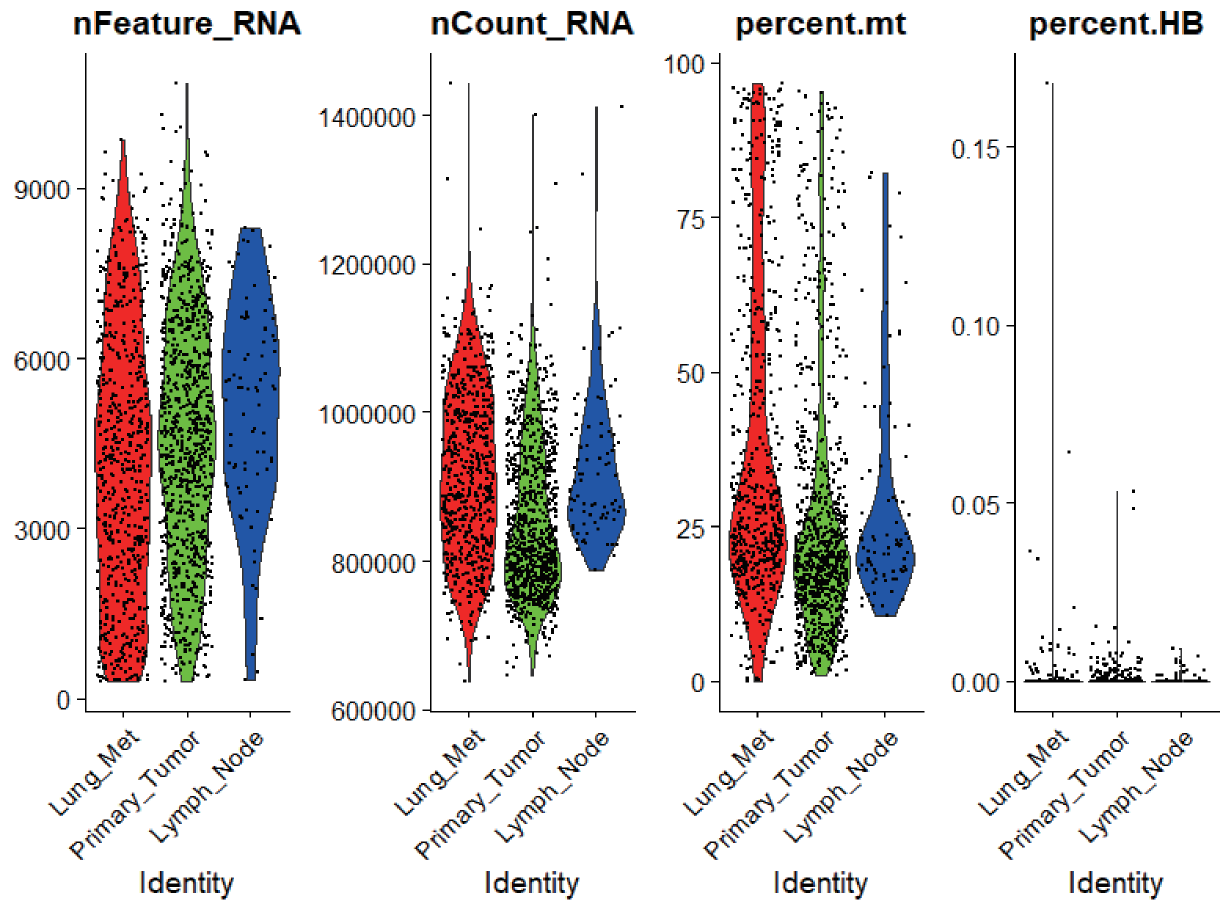

Supplemental Fig. S3 The GSE123837 dataset was retrieved, and general data were displayed in detail and grouped by tumor origin. Accordingly, the number of features, the number of counts, the percentage of mitochondrial genes, and the percentage of erythrocyte genes were listed.

Sup.
Figure S4

(A)

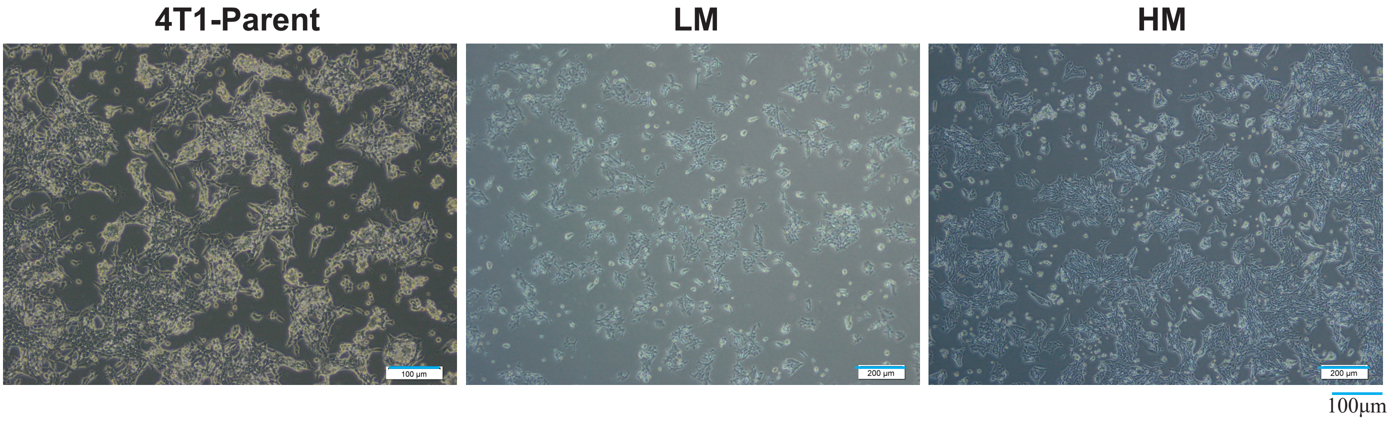

(B)

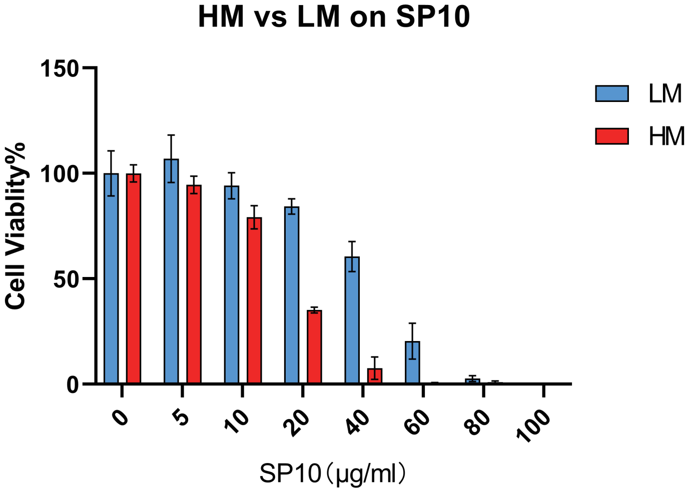

Supplemental Fig. S4 (a) Morphology of 4T1-parent cells and 4T1-LM and 4T1-HM single clones under microscope. (b) 4T1-HM and 4T1-LM were treated for 48h with iron chelators. SP10 was used. Cell viability was determined via Cell Proliferation Kit II (XTT). Cell viability in the absence of treatment was set at 100%. The student's t test with Welch's correction was utilized to compare two groups, NS represented not significant, *p**<.05, *p***<.01, *p****<.001 and Data shown as the mean ± S. E. M.

Sup.
Figure S5

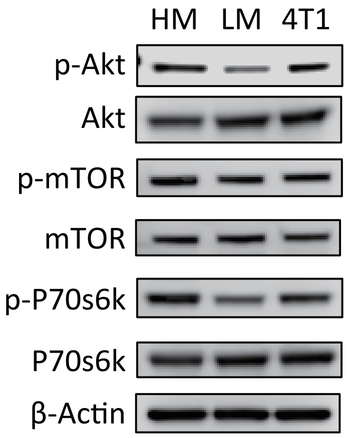

Supplemental Fig. S5 The whole cell lysis buffer of 4T1-HM, 4T1-LM, and 4T1 parent cells were collected, and the protein expression level was detected by western blot. β-actin was used as an internal control.

Sup.
Figure S6

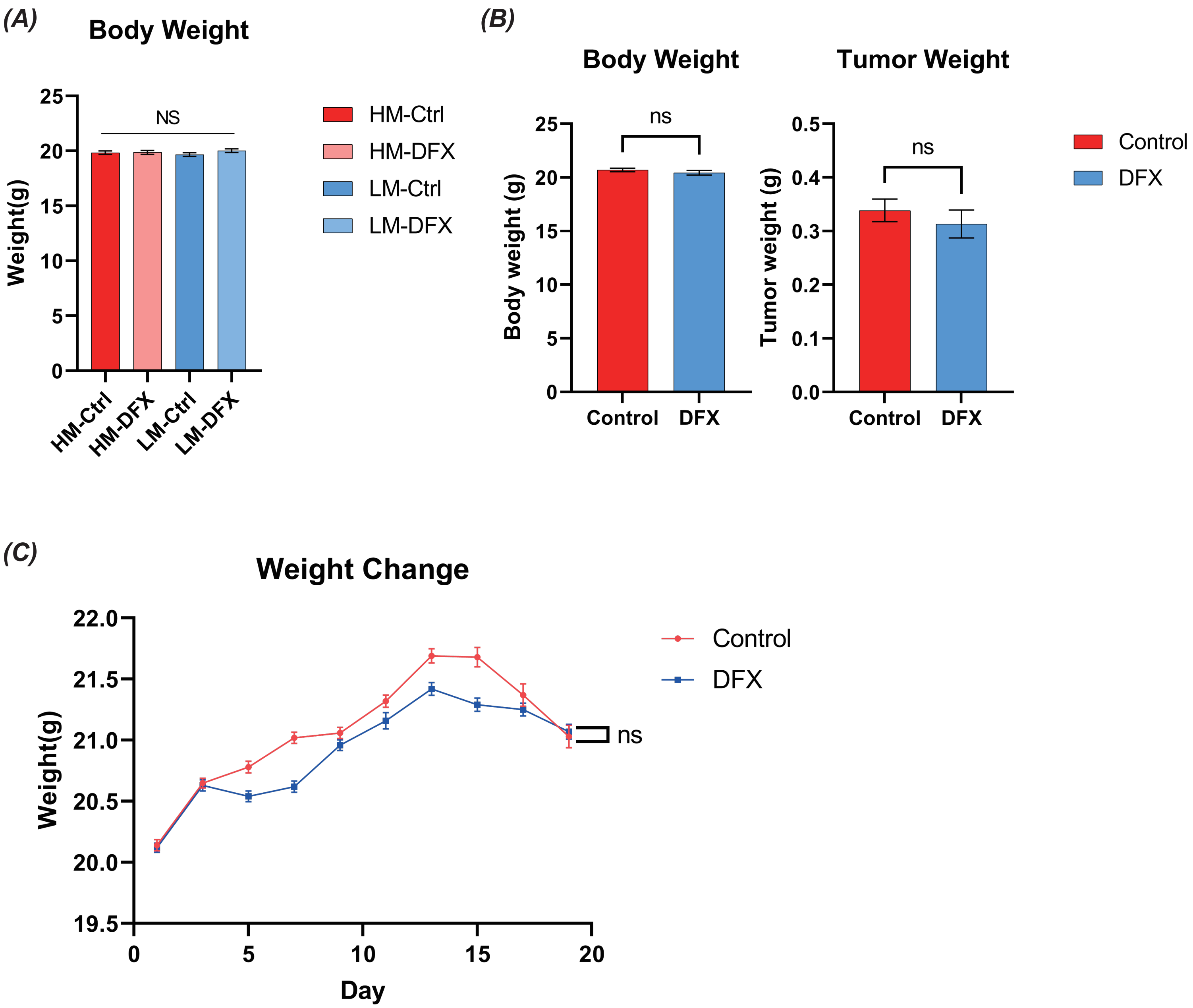

Supplemental Fig. S6 (a) Body weight of every two groups were compared with the student's t test, NS represented not significant, and Data shown as the mean ± S. E. M. (b) Body weight before surgery and resected tumor weight of two groups were compared using the student's t test, NS represented not significant, and Data shown as the mean ± S. E. M. (c) Body weight of two groups were compared with the student's t test, NS represented not significant, and Data shown as the mean ± S. E. M.
