# Supplementary material for: Highly Metastatic Subpopulation of TNBC Cells Has Limited Iron Metabolism and Is a Target of Iron Chelators
Source: Cancers (Basel). 2023 Jan 12;15(2):468. doi: 10.3390/cancers15020468 (PMC9856351; doi:10.3390/cancers15020468)
Supplement: Supplementary file 1 [file cancers-15-00468-s001.zip › Table S1.pdf]

Table S1. Antibodies applied in this study

Western blot		
**Primary antibody**		
Protein name	Dilution	Company
Cleaved-Parp	1:1000	CST #5625
c-Myc	1:1000	CST #5605
Klf4	1:1000	CST #4038
Phospho-AKT	1:1000	CST #9271
AKT	1:1000	CST #4691
Phospho-mTOR	1:1000	CST #5536
mTOR	1:1000	CST #2983
Phospho-p70S6K	1:1000	CST #9234
p70S6K	1:1000	CST #9202
β-actin	1:5000	Sigma #A5441
GAPDH	1:5000	CST #5174
**Secondary antibody**		
Goat anti-rabbit IgG HRP	1:2000	CST #7074
Goat anti-mouse IgG HRP	1:2000	CST #91196

CST(Cell Signaling Technology, Danvers, Massachusetts)
Abcam(Abcam, Cambridge, UK)
Sigma(Sigma Corporation, Setagaya, Japan)
Bioss(Bioss, Boston, Massachusett)
