# Supplementary material for: Highly Metastatic Subpopulation of TNBC Cells Has Limited Iron Metabolism and Is a Target of Iron Chelators
Source: Cancers (Basel). 2023 Jan 12;15(2):468. doi: 10.3390/cancers15020468 (PMC9856351; doi:10.3390/cancers15020468)

Fig 5 Akt-mTOR targets

p-Akt

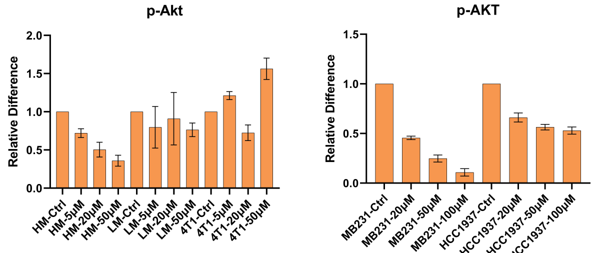

Akt

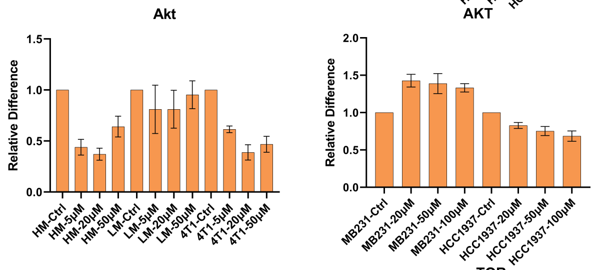

p-mTOR

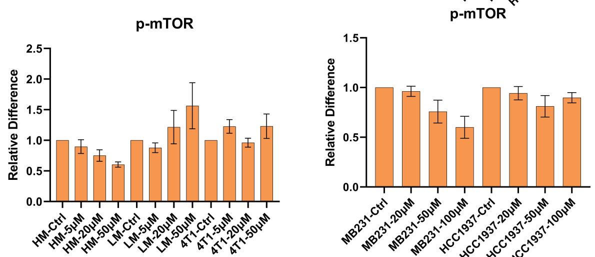

mTOR

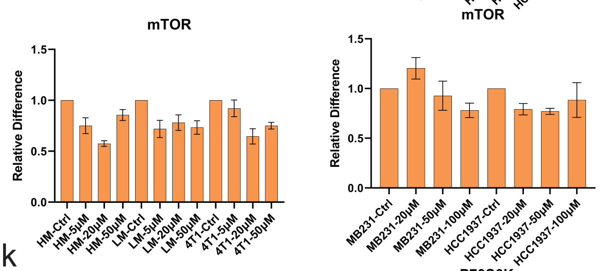

p-P70s6k

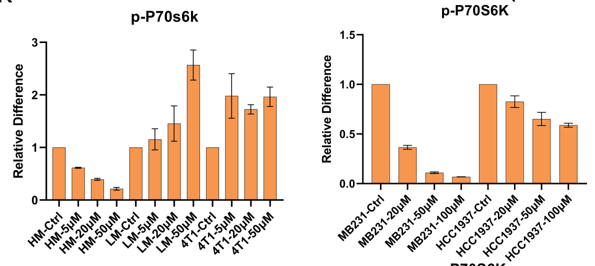

P70s6k

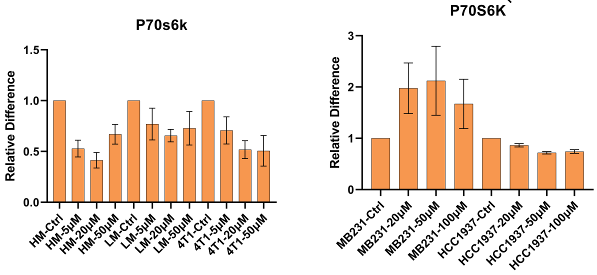

Fig 5H mTOR

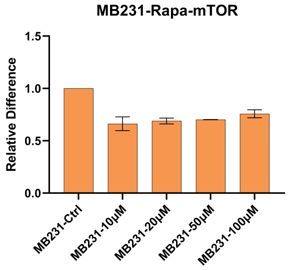

Supp Fig5 p-Akt

Akt

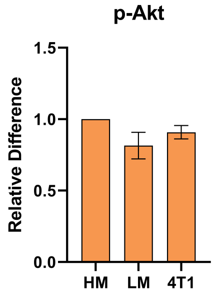

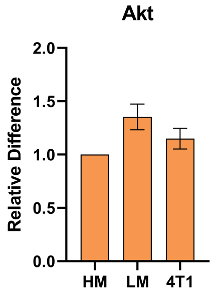

p-mTOR

mTOR

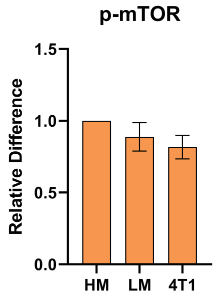

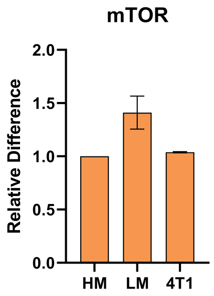

p-P70s6k

P70s6k

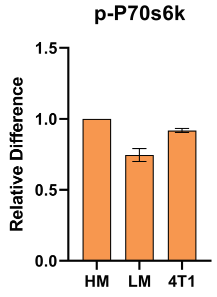

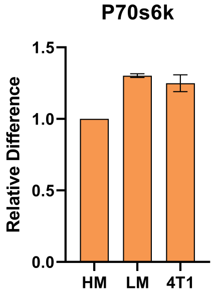

Fig 5H p-mTOR

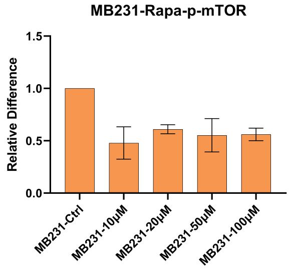

Supplement: Supplementary file 1 [file cancers-15-00468-s001.zip › WB histogram.pdf]
