# Supplementary material for: Highly Metastatic Subpopulation of TNBC Cells Has Limited Iron Metabolism and Is a Target of Iron Chelators
Source: Cancers (Basel). 2023 Jan 12;15(2):468. doi: 10.3390/cancers15020468 (PMC9856351; doi:10.3390/cancers15020468)

Fig 5A p-Akt

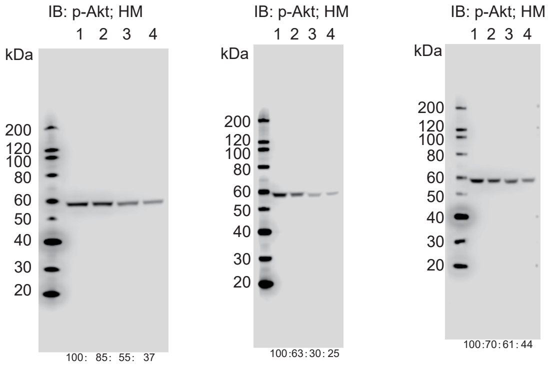

Akt

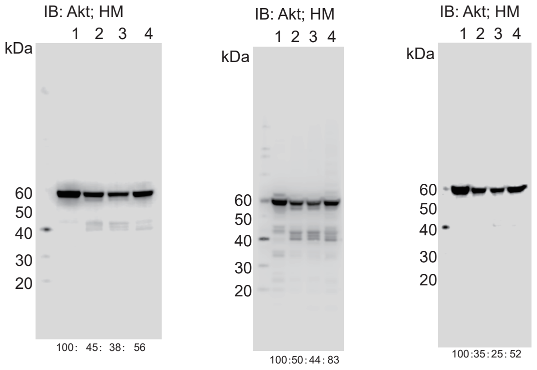

p-mTOR

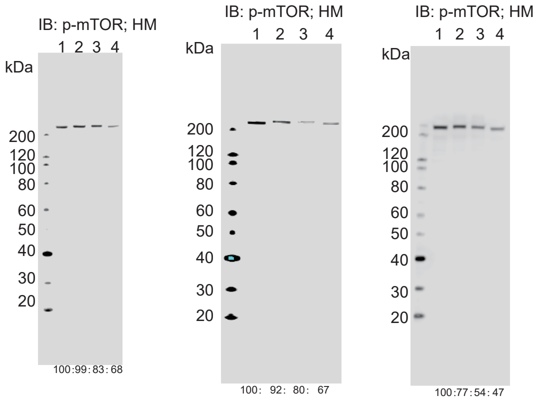

mTOR

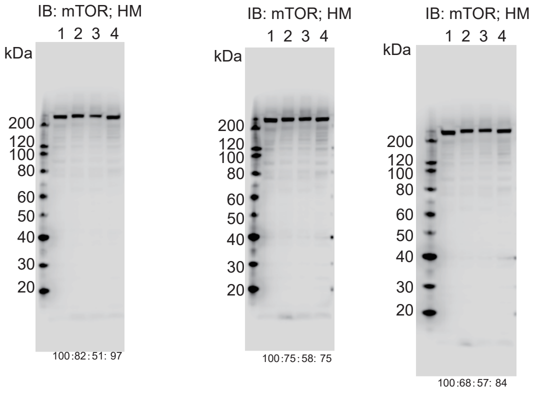

p-P70s6k

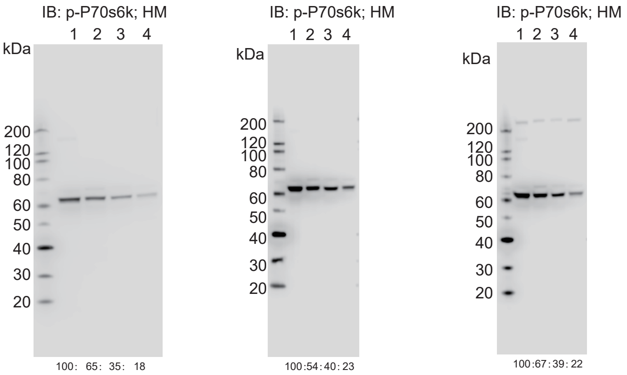

P70s6k

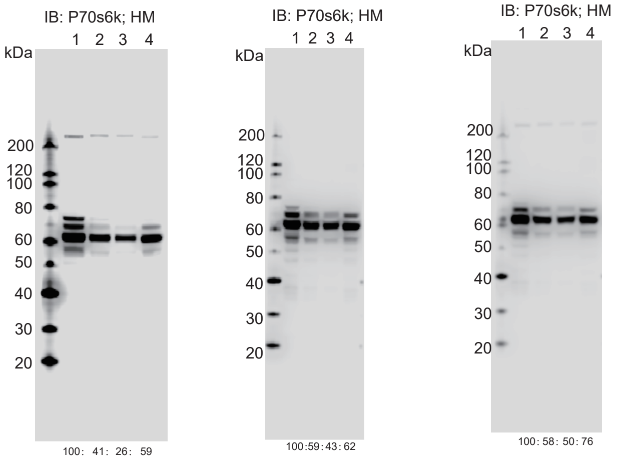

Beta-Actin

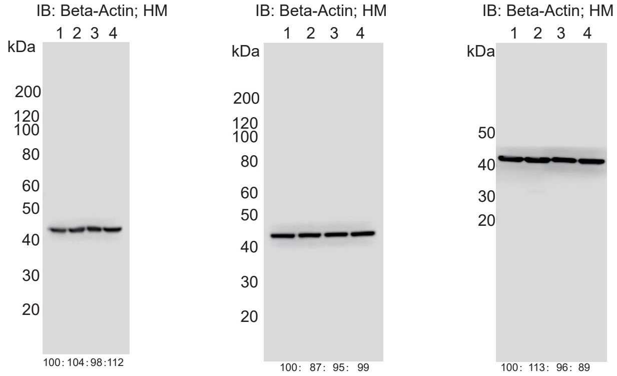

Fig 5B p-Akt

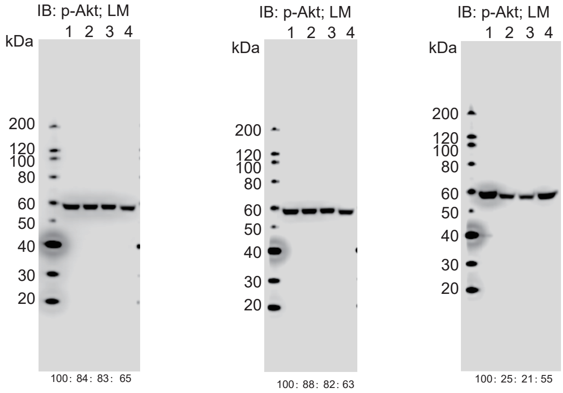

Akt

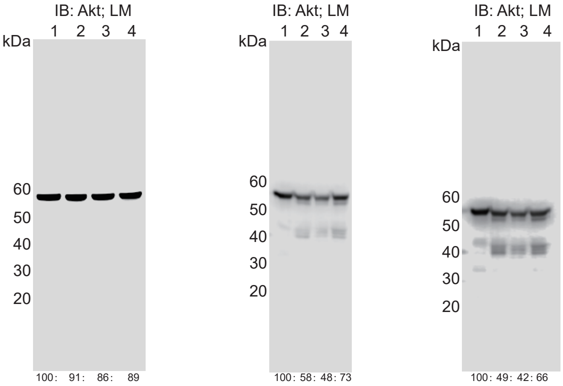

p-mTOR

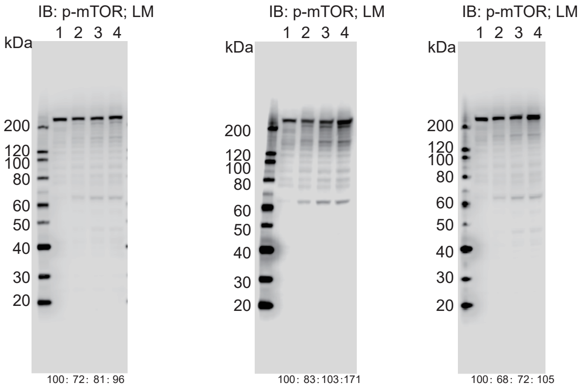

mTOR

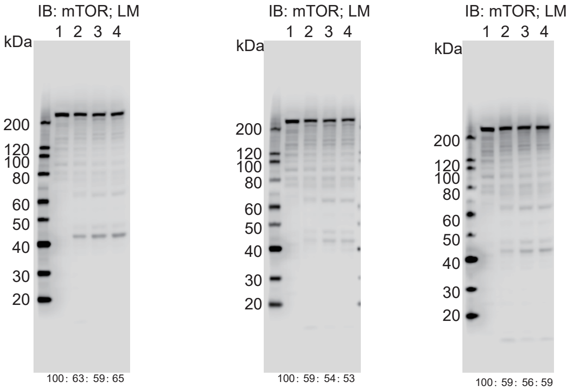

p-P70s6k

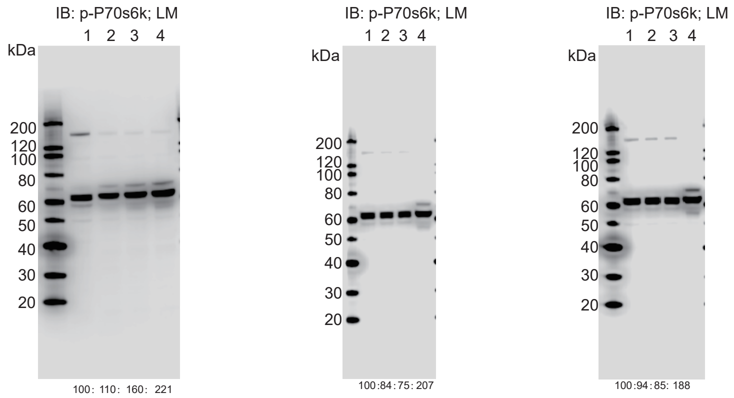

P70s6k

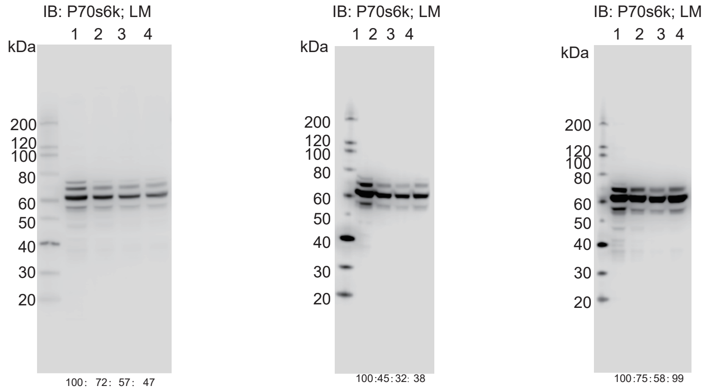

Beta-Actin

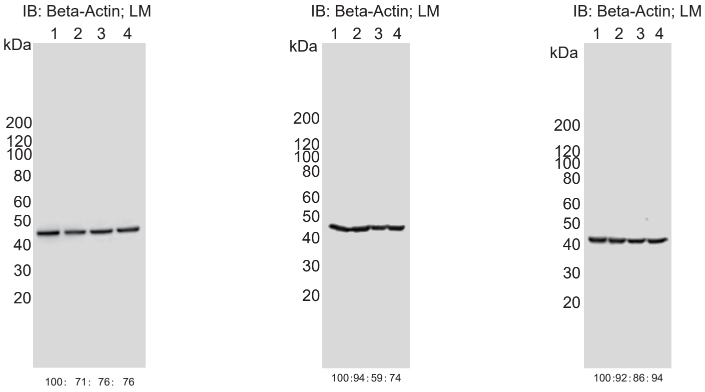

Fig 5D p-AKT

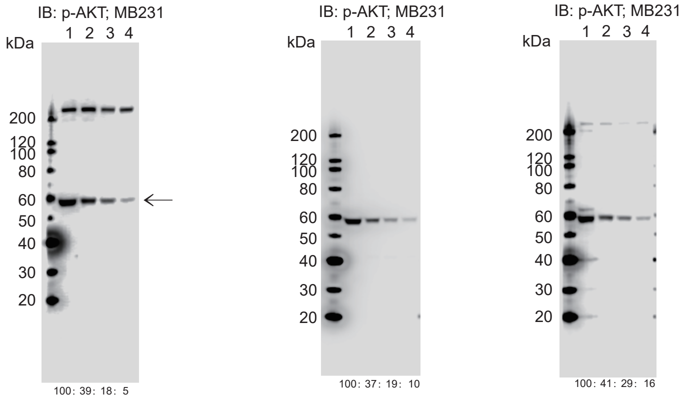

AKT

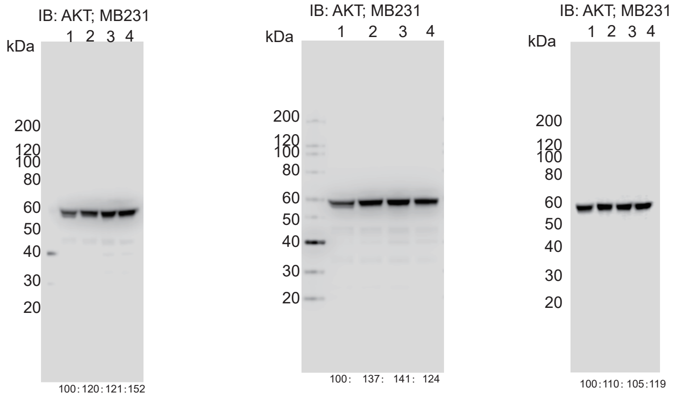

p-mTOR

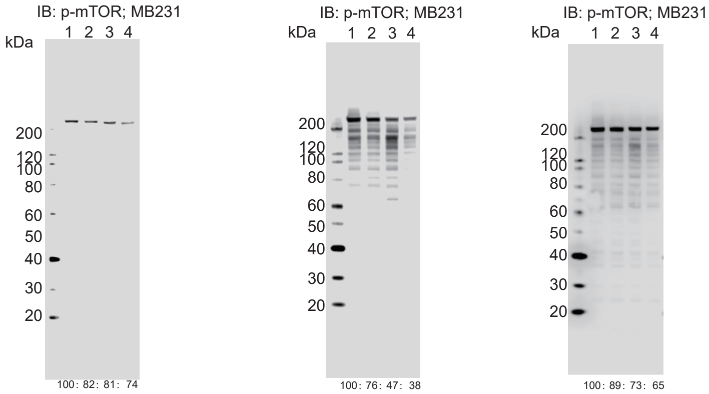

mTOR

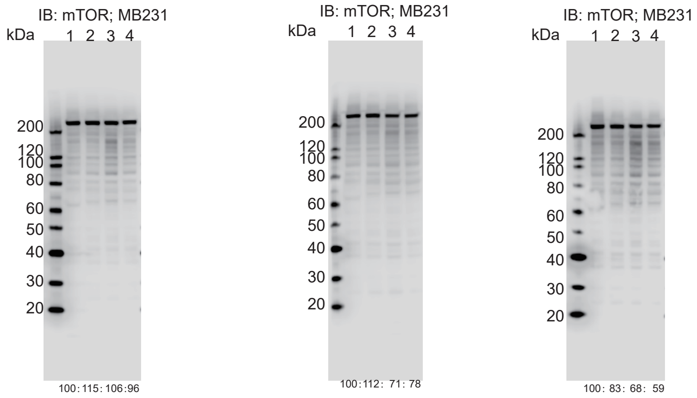

p-P70S6K

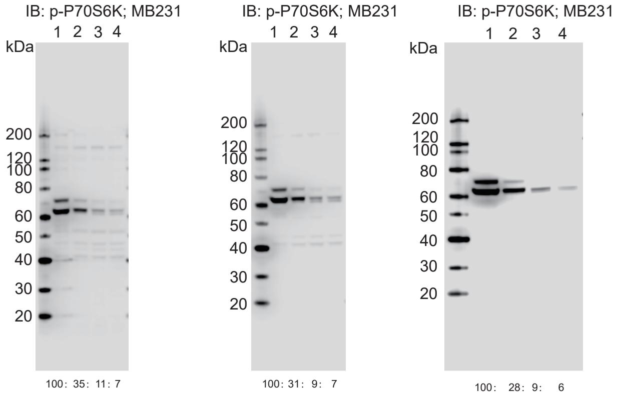

P70S6K

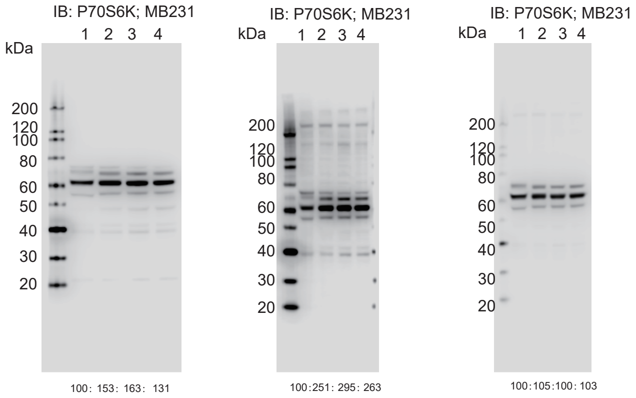

Beta-ACTIN

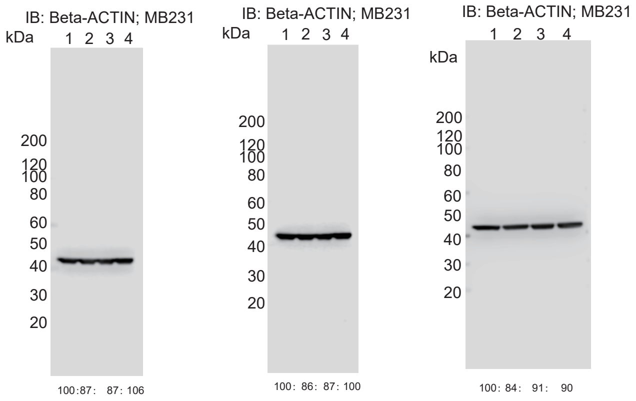

Fig 5E p-AKT

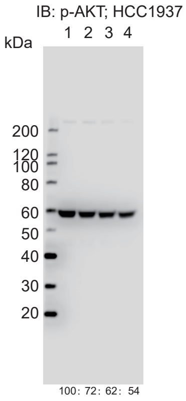

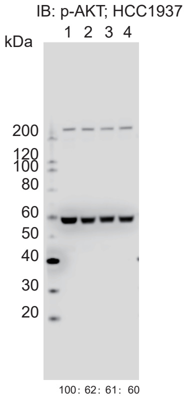

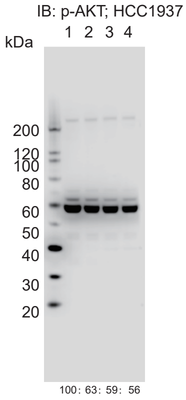

AKT

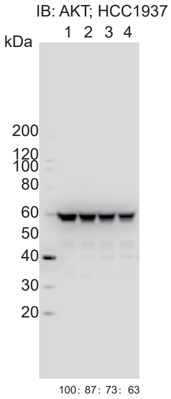

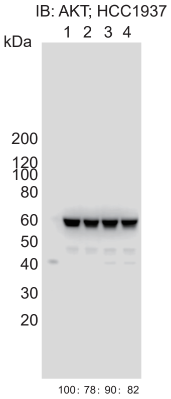

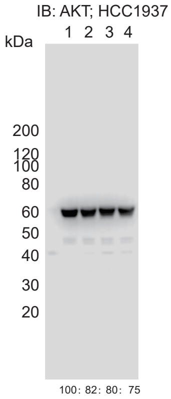

p-mTOR

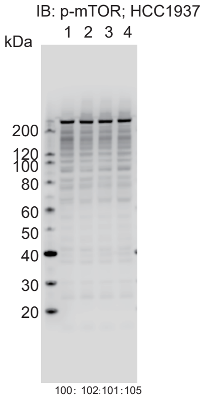

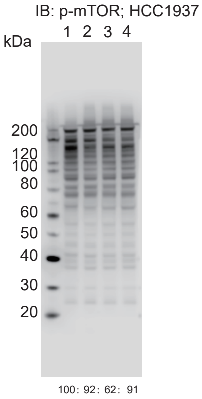

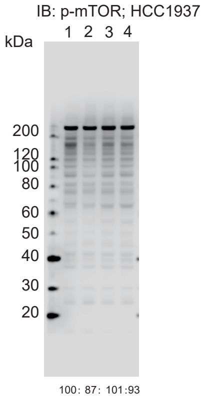

mTOR

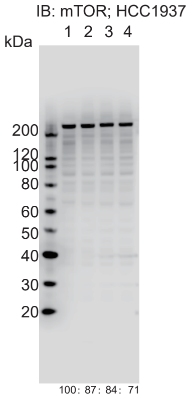

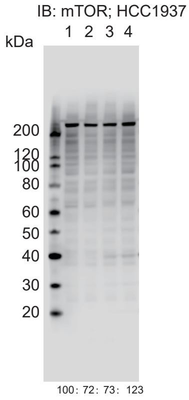

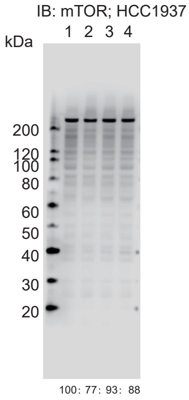

p-P70S6K

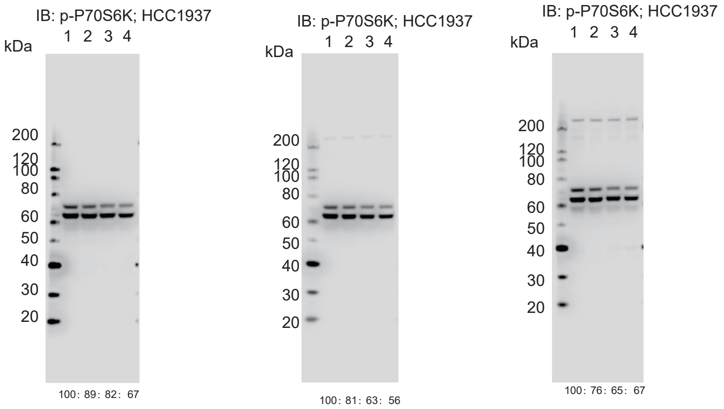

P70S6K

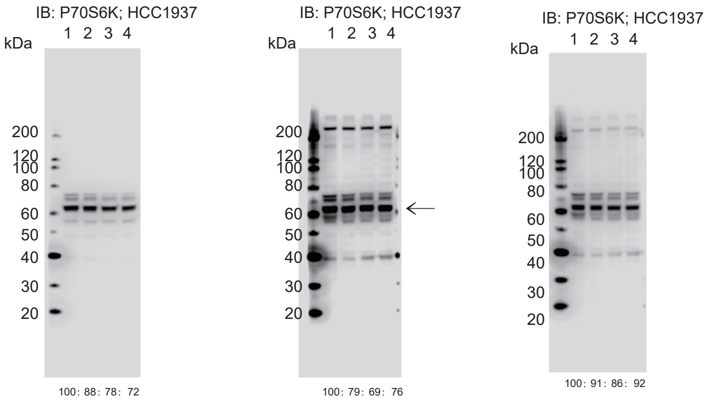

Beta-ACTIN

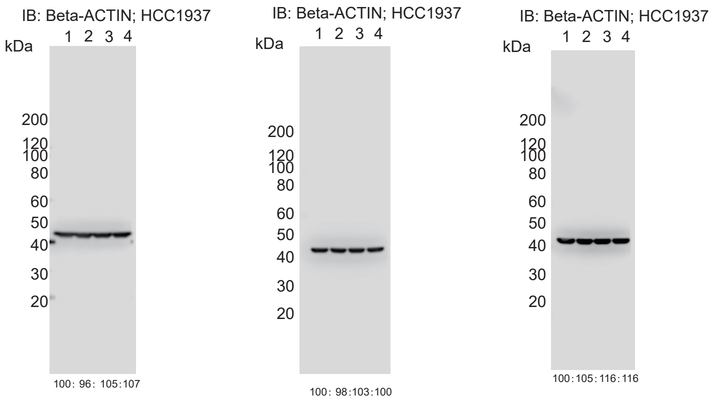

Fig 5G p-mTOR

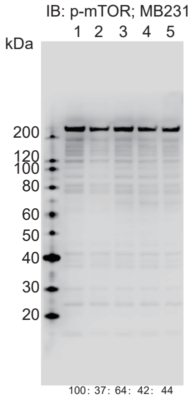

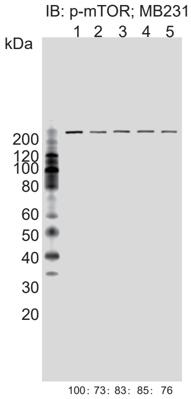

mTOR

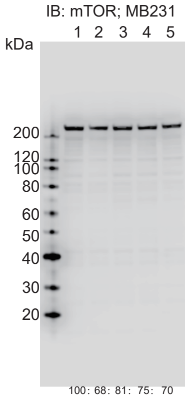

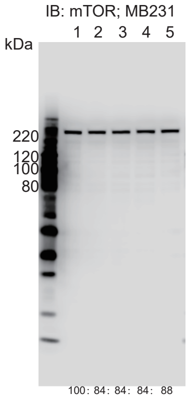

Beta-ACTIN

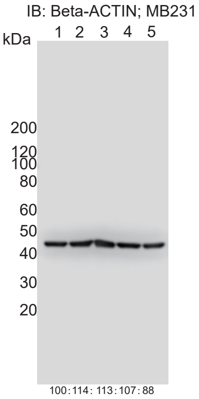

Fig 6A Cleaved and non-Cleaved Parp

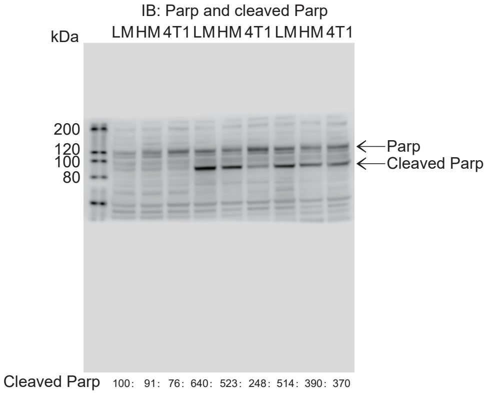

c-Myc

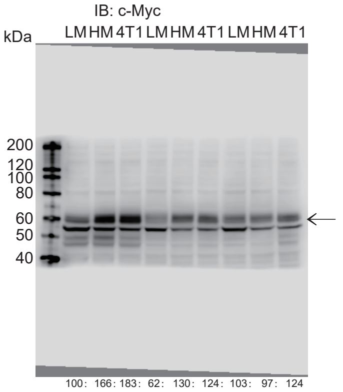

Klf4

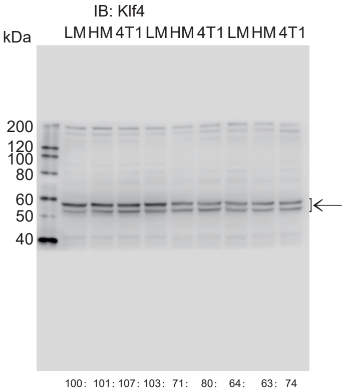

Gapdh

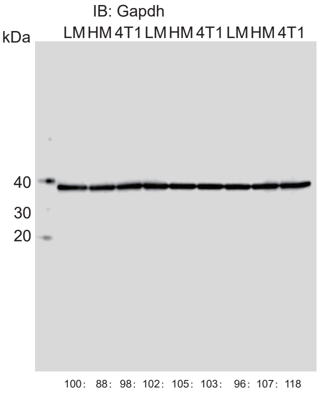

Supp. Fig 5 p-Akt

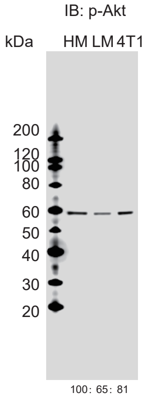

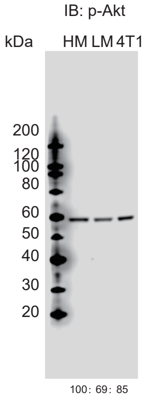

Akt

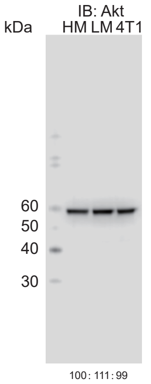

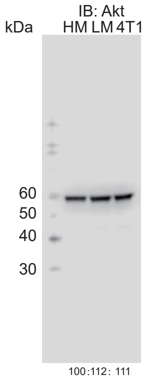

p-mTOR

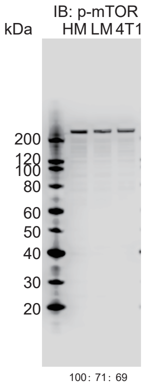

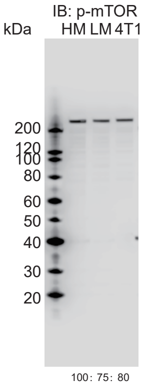

mTOR

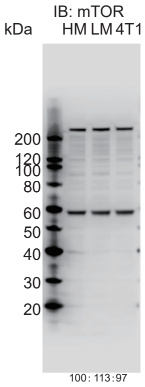

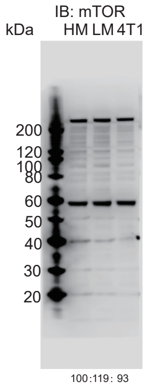

p-P70s6k

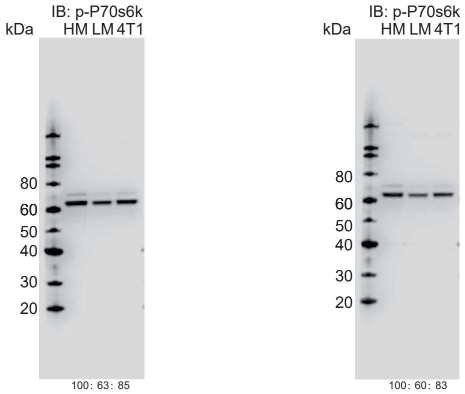

P70s6k

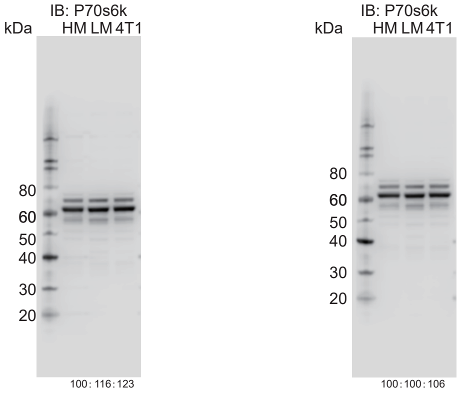

Beta-Actin

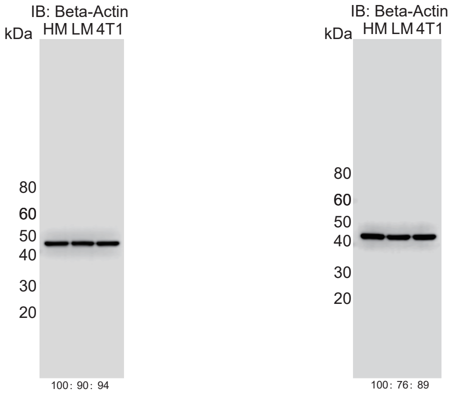

Supplement: Supplementary file 1 [file cancers-15-00468-s001.zip › WB Whole blotting.pdf]
